# Modelling the effect of compliance with WHO salt recommendations on cardiovascular disease mortality and costs in Brazil

**DOI:** 10.1371/journal.pone.0235514

**Published:** 2020-07-09

**Authors:** Eduardo Augusto Fernandes Nilson, Adriana Blanco Metlzer, Marie-Eve Labonté, Patrícia Constante Jaime

**Affiliations:** 1 Department of Global Health and Sustainability, School of Public Health, University of Sao Paulo, Sao Paulo, São Paulo, Brazil; 2 Costa Rican Institute of Research and Training in Nutrition and Health (INCIENSA), San Jose, Costa Rica; 3 Institute of Nutrition and Functional Foods, Laval University, Québec City, Quebec, Canada; 4 Department of Global Health and Sustainability, School of Public Health, University of Sao Paulo, Sao Paulo, São Paulo, Brazil; International University of Health and Welfare, School of Medicine, JAPAN

## Abstract

**Introduction:**

Cardiovascular diseases (CVDs) represent the main cause of death among non-communicable diseases (NCDs) in Brazil, and they have a high economic impact on health systems. Most populations around the world, including Brazilians, consume excessive sodium, which increases blood pressure and the risk of CVDs.

**Objective:**

To model the estimated deaths and costs associated with CVDs, which are mediated by increased blood pressure attributable to excessive sodium consumption in adults from the perspective of the Brazilian public health system in 2017.

**Methods:**

We employed two macrosimulation methods, using top-down approaches and based on the same relative risks. The models estimated the mortality and costs-of-illness attributable to excessive sodium intake and mediated by hypertension for adults aged over 30 years in 2017. Direct healthcare cost data (inpatient care, outpatient care and medications) were extracted from the Ministry of Health information systems and official records.

**Results:**

In 2017, an estimated 46,651 deaths from CVDs could have been prevented if the average sodium consumption had been reduced to 2 g/day in Brazil. Premature deaths related to excessive sodium consumption caused 575,172 Years of Life Lost and US$ 752.7 million in productivity losses to the economy. In the same year, the National Health System’s costs of hospitalizations, outpatient care and medication for hypertension attributable to excessive sodium consumption totaled US$192.1 million. The main causes of death and costs associated with CVDs were coronary heart disease and stroke, followed by hypertensive disease, heart failure and aortic aneurysm.

**Conclusion:**

Excessive sodium consumption is estimated to account for 15% of deaths by CVDs and to 14% of the inpatient and outpatient costs associated with CVD. It also has high societal costs in terms of premature deaths. CVDs are a leading cause of disease and economic burden on the global, regional and country levels. As a largely preventable and treatable conditions, CVDs require the strengthening of cost-effective policies, supported by evidence, including modeling studies, to reduce the costs relating to illness borne by the Brazilian public health system and society.

## Introduction

Non-communicable diseases (NCDs) are the main cause of death and disability globally and in Brazil. According to the Global Burden of Disease studies, in 2017, 11 million deaths and 255 million Disability Adjusted Years (DALYs) were attributable to dietary risk factors worldwide. A high intake of sodium is considered the primary diet-related risk factor (being responsible for 3 million deaths and 70 million DALYs), because of its strong association with NCDs, such as cardiovascular diseases (ischemic heart disease, stroke, and hypertension) [1].

Cardiovascular diseases are one of the leading causes of global mortality and morbidity, and they also represent a major economic burden on health care systems in terms of the direct (e.g., medical consultations, hospitalizations, rehabilitation services, and drugs) and indirect (e.g., losses of productivity due to premature mortality and short- or long-term disability) costs associated with mortality and morbidity.

The costs of CVDs, as NCDs, are likely to continue to grow due to inadequate diets, an increase in obesity and the aging of societies, so it is very important to address the burden of these diseases in terms of mortality, morbidity and the costs to health systems and society. In addition, economic studies can provide evidence to support CVD prevention programs, which are very cost-effective measures, compared to the costs associated with treating CVD-related conditions [2].

In recent decades, the development of many health and economic modelling methodologies, involving both macro and microsimulations and static and dynamic analysis has improved and supported policy decisions by analyzing the potential impact and cost-effectiveness of health interventions. Most models focused on ex ante policy estimations and scenario comparisons for obesity and NCDs and often incorporated economic analysis in traditional mortality and morbidity outcomes [3][4][5][6]. Choosing an epidemiological model for health and economic evaluation depends on factors, such as the data availability, timeframe for results, programming and computer capacity, but most models are based on common principles, such as the use of relative risks, drawn from the literature, to establish causal frameworks that link changes in risk factors with health and cost outcomes [7].

For example, the health scenario static model for NCD risk factors, called PRIME (Preventable Risk Integrated ModEl), was developed by the University of Oxford and adapted methodologies similar to those used by the Global Burden of Disease Project. The model compares deaths from NCDs using a baseline and counterfactual scenarios and changing risk factors, such as diet, physical activity, alcohol consumption and smoking [8]. PRIME has been used for the evaluation of several policy scenarios in the United Kingdom, other European countries and Canada [9] and is a very comprehensive and user-friendly methodology for comparing policy options and estimating the impact of risk factor changes in terms of counterfactual scenarios.

Economic studies have highlighted the burden of NCDs, especially CVDs, on countries and health systems. In most low and middle income countries, the annual cost of CVD care is many times the total health expenditure per capita [5]. In Mexico, the burden of cardiovascular diseases comprises approximately 4% of the total national healthcare expenditure and approximately 46% of the health system cost [10]. In the United States, cost estimates of CVD episodes are at least twice as high as the cost of some conditions in the European Union [11].

In Brazil, the first national salt/sodium consumption based on spot urinary sodium excretion was released in 2019, despite data collection was conducted in 2013. The average salt consumption was estimated in 9.34g/day among adults (9.63g for men and 9.08g for women) and 97.6% of the adults consumed over the WHO recommendation of 5g/day [12]. The main dietary sources of sodium, according to national food acquisition surveys, are salt added to foods and industrialized products, as well as foods consumed within and out of the household [13]. As a result, 22.8% of the Brazilian adult population are hypertensive (25.8% of men and 20.0% of women) [14].

Consequently, salt/sodium reduction and hypertension prevention and control require multiple strategies, from health education and nutritional counseling to food reformulation, along with the secondary prevention and treatment of hypertension and CVD. The comparative impact of these conditions on health and costs is still unknown [15].

According to the 2013 Brazilian National Health Survey, chronic diseases increased hospitalizations and compromised normal activities among adults. Non-communicable diseases were also associated with more medical consultations and a greater use of the health services [16].

In Brazil, most of the population depends on the National Health System (SUS) for health care, which is publicly funded and universal, from primary health care to hospital treatment. Despite the high cost of CVD, there are few Brazilian studies that have estimated the federal government expenditures on the mortality and economic burden of these diseases and their major risk factor, excessive sodium consumption [17]. Nevertheless, cost-of-illness methodologies have been successfully used to estimate the costs associated with obesity and chronic kidney disease [18][19].

Considering the scarcity of data on the economic burden of dietary risk factors, such as sodium consumption, on cardiovascular disease, the present study aims to estimate the burden of cardiovascular diseases attributable to excessive sodium consumption, with hypertension as the intermediate outcome, in terms of the deaths and costs to the Brazilian publicly funded health system in 2017. The findings of this study should contribute to the improvement of the management of health system expenditures in developing countries, including Brazil.

## Materials and methods

### Study setting

This study provides, first, a health impact evaluation, which models the effect of sodium reduction on mortality associated with blood pressure and CVD, followed by a cost-of-illness analysis [14], from the perspective of the Brazilian National Health System (Sistema Único de Saúde, SUS), in 2017. All input variables and data sources are summarized in Table 1.

**Table 1:** Summary of the key model inputs and sources for health and economic modeling.

Model inputs	Value	Source
Baseline characteristics		
Demographics		Brazilian Population Estimates (IBGE) [20]
Salt consumption		National Health Survey (IBGE) 2013 [12]
Deaths by CVD		SIM 2017 [21]
Hospitalization costs		SIH-SUS 2017 [22]
Outpatient costs		SIA-SUS 2017 [23]
Workforce characteristics		Continuous National Household Sample Survey–IBGE [24]
Effect of salt consumption on systolic blood pressure	-5.80 (-2.50, 9.20)	[25]
Relative risk of systolic blood pressure	Unit of change: 20 mmHg SBP decrease	[26]
Coronary heart disease	<49 y: 0.49 (0.45–0.53)	[26]
	50–59 y: 0.50 (0.49–0.52)	
	60–69 y: 0.54 (0.53–0.55)	
	70–79 y: 0.60 (0.58–0.61)	
	Over 79 y: 0.67 (0.64–0.70)	
Stroke	<49 y: 0.36 (0.32–0.40)	[26]
	50–59 y: 0.38 (0.35–0.40)	
	60–69 y: 0.43 (0.41–0.45)	
	70–79 y: 0.50 (0.48–0.52)	
	Over 79 y: 0.67 (0.63–0.71)	
Hypertensive disease	0.22 (0.20–0.25)	[26]
Heart failure	0.53 (0.48–0.59)	[26]
Pulmonary embolism	0.72 (0.60–0.87)	[26]
Rheumatic heart disease	0.74 (0.61–0.89)	[26]
Aortic aneurysm	0.55 (0.49–0.62)	[26]

The 1988 Brazilian Constitution states health as a citizen’s right and a duty of the state, and it established the foundations for the Unified Health System (SUS) and its main principles of universality, integrality, and social participation [27]. SUS is responsible for approximately 75% of medical care in Brazil and for health promotion, health surveillance, vector control, health education, and primary, specialist outpatient, and hospital level health care [18].

Brazil’s Information System on Mortality (*Sistema de Informações de Mortalidade*, SIM) is a comprehensive, population-based system, with high-quality information on mortality by cause, sex, education, age-group and location. Regarding the costs of disease treatment to the national health system, all costs associated with secondary health care are registered by public and private hospitals in the SUS Outpatient Information System (*Sistema de Informação Ambulatorial*, SIA/SUS) and the Hospital Information System (*Sistema de Informação Hospitalar*, SIH/SUS).

The costs of anti-hypertension drugs provided by the Farmacia Popular Program were obtained from the National Department of Pharmaceutical Care. The “Farmácia Popular do Brasil” Program (PFPB) was established through a public owned pharmacy network, and was subsequently expanded through partnerships with the retail private pharmacies, especially providing medications for hypertension, diabetes and asthma [28].

The most recent nationally representative salt consumption data was assessed from spot urine collected in 2013, as part of the Brazilian National Health Survey (NHS), a household survey with a representative sample of the Brazilian adult population. The estimated salt intake was described according to gender (male and female), age group (18 to 29; 30 to 44; 45 to 59; and 60 or older), among other disaggregations. We have assumed that salt intake remained constant from 2013 to 2017, considering that, in the previous decade, despite changes in dietary salt sources, the estimates of total salt intake was unchanged [13].

The present study ran a non-communicable disease scenario macro-simulation models to estimate the potential impact of modifications in dietary salt intakes on mortality and costs from cardiovascular diseases (CVD). A detailed presentation of the data sources and the methods used is available as Supplementary Materials.

Cardiovascular disease is considered, in this study, to comprise coronary heart disease, stroke, hypertensive disease, heart failure, aortic aneurysm, pulmonary embolism and rheumatic heart disease (International Codes of Disease (ICDs): I20–25, I60–69, I10–15, I50, I71, I26 and I05–09, respectively).

### Baseline and counterfactual scenarios

The observed salt intake from the Brazilian National Health Survey of 2013 was used to determine the reference (baseline) scenario and the counterfactual scenario considered that average salt consumption was reduced to 5g/day.

### Health impact modeling

A comparative risk assessment model, the Preventable Risk Integrated ModEl (PRIME) was used to estimate the deaths from cardiovascular diseases that could be averted or postponed based on the sodium recommendation scenario (sodium intake of 2g/day in the Brazilian population) compared to the baseline scenario [8].

PRIME is designed to estimate the impact of changes in the age- and sex-specific distribution of one or more out of twelve behavioural risk factors covering diet, physical activity, alcohol consumption and tobacco consumption on NCD mortality, through direct associations or through mediating factors that include BMI, blood pressure and blood cholesterol. Only sodium intake was evaluated in the present study. PRIME parameterizes the risk factors and NCD mortality based on results of published meta-analyses of epidemiological studies and includes parameters which have been appropriately adjusted for other behavioural risk factors in order to minimize the risk of double counting of effect size [8].

The PRIME application is available from the University of Oxford upon request and its data requirements include: 1) age- and sex-specific estimates of the annual number of deaths from each relevant NCD in the population under study; 2) age and sex-specific estimates of the number of individuals living in the population; 3) the baseline distribution of behavioural risk factors in the population of interest (herein, sodium intake under the baseline scenario); and 4) the counterfactual distribution of the variables of interest (herein, sodium intake under the counterfactual recommendation scenario).

The estimation of the impacts of sodium consumption on cardiovascular disease outcomes in PRIME is based on the parametrization of sodium intake and changes in systolic blood pressure [25] and of changes in blood pressure and cardiovascular outcomes [29]. Firstly the model parametrizes the changes in sodium consumption (converted to the equivalent salt intake) and its effects on systolic blood pressure, creating a linear estimation of increase in SBP for each gram of salt added to the diet and, afterwards, it parametrizes these 1g salt intervals for a combined relative risk related to cardiovascular diseases [8].

In the present study, data on sodium consumption, mortality from CVDs and population demographics were obtained, respectively, from publicly available tables of the National Health Survey–PNS 2013 [12], Brazilian Mortality Information System (SIM) for 2017 [21] and the Brazilian Institute of Geography and Statistics (IBGE) for 2017 [20]. Demographic and mortality data were stratified by gender and 5-year age bands, and mortality data were based on the World Health Organization (WHO) International Classification of Diseases 10 (ICD 10)

Probabilistic sensitivity analysis for obtaining the 95% Uncertainty Intervals (UIs) were based the results generated from 10,000 iterations of a Monte Carlo analysis built in PRIME, in which the estimates of relative risks used to parameterize the model were allowed to vary randomly according to the distributions described in the literature. The framework of these macrosimulations allows stochastic uncertainty, parameter uncertainty, and heterogeneity to be reflected in the reported UI.

### Cost-of-illness modeling

#### Costs of premature deaths

The estimated Years of Life Lost (YLL), which are part of the estimates for DALYs (Disability Adjusted Life Years), were calculated using the formula used by GBD: YLL = N x L, where: N = the number of deaths from CVDs averted or postponed (estimated through PRIME) and L = the standard life expectancy at the age of death in years for the Brazilian Population [30].

The Years of Productive Life Lost (YPLL) were estimated through the Human Capital Approach [31], which calculates the present value of potential time in the workforce (the measure of productivity) using country-specific data for 2017. YPLL was calculated by multiplying the YLL from age 15 to the pension age (60 years for women, and 65 years for men) by the average national wage and the labor force participation estimates from the Continuous National Household Sample Survey (PNAD), provided by IBGE [24].

#### Direct costs of disease to the Brazilian Health System

To calculate the attributable costs to sodium consumption, the cost-of-illness model used in this work [32] is based on the findings of the meta-analysis of randomized controlled trials, which established the age-specific relationship between salt and blood pressure [25], and other meta-analysis that link hypertension with cardiovascular disease [26], similarly to PRIME.

Salt consumption is considered a continuous risk factor and the relative risks are parameterized in order to describe the change in risk for a unit decrease in the risk factor (salt consumption) across a given range. First, regarding the impact of salt/sodium consumption on blood pressure, the model considers that the reduction of salt consumption by 6g/day was associated with a reduction of 5.8mmHg in systolic blood pressure (SBP) [25]. Then, the differential SBP (mmHg) from salt is estimated, considering intervals of consumption, ranging from less than 5g to over 12g, and adjusted to the relative risks for changes in SBP and each CVD outcome, by age group and sex [26], in order to estimate relative risks for each CVD for unit of change in salt consumption.

The estimated cost of CVDs associated with excessive sodium consumption was calculated through the population attributable risk (PAR) by sex and age and multiplied by the costs associated with hospitalizations, outpatient care and medications for hypertension. Data on costs are publicly available from the records in the SUS Outpatient Information System (SIA/SUS), in the Hospital Information System (SIH/SUS) and from the Farmacia Popular Program, from January 2017 to December 2017. The population considered for analysis was restricted to people over 30 years of age.

PAR was calculated by the following formula [33]:

PAR=100xP(RR−1)/(P(RR−1)+1)

Where:

P = The prevalence of excessive sodium consumption, as obtained from microdata from the Household Budget Survey, and

RR = The combined relative risk of sodium consumption leading to increased blood pressure, and blood pressure leading to CVD outcomes, as used in the PRIME model for mortality estimations [8].

A top-down approach was adopted for the identification of the direct costs of CVDs by specific ICD codes and valuations, based on administrative data obtained from the SIA/SUS and SIH/SUS. These systems serve as a registry of all procedures, for which the Brazilian Ministry of Health reimburses health facilities (hospitals, clinics, and laboratories), public or private, and provide services to the Unified Health System. Direct costs were defined as those of outpatient (SIA/SUS) and inpatient (SIH/SUS) procedures, such as doctor’s appointments, laboratory tests, medications, hospital admissions, treatment of complications, renal replacement therapy, and renal transplantation. Non-medical direct costs (patient transport and caregiver payment), indirect costs (absenteeism, presentism, and early death), and intangible costs (loss of ability to work, loss of quality of life, etc.) were disregarded, except for the costs of premature deaths.

Nominal values for 2017 were used, without any adjustment for inflation. This is a common practice for Brazilian studies of public health costs, which use administrative data, because of the lack of a regular fee schedule of procedures, offered by the Unified Health System. Costs were collected in Brazilian Reals (R$) and subsequently converted to U.S. dollars (US$), at an exchange rate of US$1 = R$3.19, current at December 31, 2017, as reported by the Central Bank of Brazil.

A probabilistic sensitivity analysis was performed via a Monte Carlo approach using the Ersatz package [34] to assess the potential uncertainty in the key model inputs, using 10,000 simulations, with 95% uncertainty intervals, based on the 2.5th and 97.5th percentiles of the simulations.

A full description of the cost-of-illness, including all the parameters and methodological steps of the model, can be found in the Supplemental Materials [32].

As this study used only aggregate, public information from government databases, which is in the public domain and offers no possibility of identifying individual subjects, approval by a review board of the National Research Ethics Committee (CEP/CONEP) was not required.

## Results

### Deaths attributable to excessive sodium consumption

In 2017, 49% of the Brazilian population was 30 years of age or older (95 million people), and according to the National Mortality Information System [21], 734,437 people in the country died from preventable diseases. Among these, 312,163 died from cardiovascular disease.

Table 2 lists the estimated number of deaths that may be averted or delayed if Brazilian men and women aged 30 years of more reduced their salt consumption to 5 g per day (2 g of sodium/day), as recommended by the WHO, the differences in salt consumption by men and women according to their age-groups.

**Table 2:** Estimated number of deaths attributed to excessive sodium consumption in Brazil, 2017.

	Number of deaths that may be averted or delayed if the average sodium consumption was reduced to 2 g/day (95% UI)		
Cause of death	Men	Women	Total
Cardiovascular disease	24,287 (10,448–37,386)	22,643 (9,618–34,426)	46,651 (20,066–71,812)
Coronary heart disease	6,961 (2,983–10,793)	5,713 (2,447–8,854)	12,677 (5,430–19,646)
Stroke	7,630 (3,259–11,762)	7,113 (3,038–10,965)	14,742 (6,297–22,727)
Heart failure	1,683 (719–2,653)	1,628 (695–2,567)	3,310 (1,414–5,220)
Aortic aneurysm	549 (232–883)	327 (138–525)	876 (370–1,408)
Pulmonary embolism	134 (42–266)	170 (54–338)	304 (96–603)
Rheumatic heart disease	53 (15–109)	86 (24–178)	139 (38–287)
Hypertensive disease	7,275 (3,199–10,921)	7,328 (3,222–11,000)	14,603 (6,422–21,921)

Considering the excessive salt consumption by Brazilians, premature deaths (<75 years) represented 53% of the deaths that may have been prevented through sodium reduction. Coronary heart disease (CHD), stroke and hypertensive disease accounted for 90% of these deaths. It is estimated that 6.4% of all preventable deaths and 15.0% of CVD deaths could be averted or delayed by consuming less salt, considering the average salt consumption at 9.34g/day.

#### Years of Life Lost (YLL)

Premature deaths are also responsible for a large burden in terms of Years of Life Lost (YLL), which are part of the Disability Adjusted Life Years (DALY). All cardiovascular deaths attributable to excessive sodium consumption, in 2017, are estimated to have caused 575,172 YLL, most of which were from CHD, stroke and hypertensive disease, as shown in Table 3. The YLL of men are, in general, higher than those of women, which is likely due to their higher rates of premature death by CVD.

**Table 3:** Estimated Years of Life Lost (YLL) due to excessive sodium consumption in Brazil, 2017.

	Years of Life Lost due to excessive sodium consumption (95% UI)		
Disease	Men	Women	Total
Cardiovascular disease	301,668 (130,282–460,696)	273,504 (118,119–417,685)	575,172 (248,401–878,381)
Coronary heart disease	87,763 (37,589–136,010)	77,798 (33,321–120,568)	165,561 (70,910–256,577)
Stroke	103,361 (44,147–159,343)	83,936 (35,850–129,396)	187,297 (79,998–288,739)
Heart failure	18,400 (7,858–29,013)	18,323 (7,825–28,892)	36,722 (15,682–57,905)
Aortic aneurysm	6,887 (2,906–11,072)	4,259 (1,797–6,846)	11,146 (4,703–17,918)
Pulmonary embolism	1,750 (553–3,481)	2,283 (722–4,540)	4,033 (1,275–8,021)
Rheumatic heart disease	756 (209–1,562)	1,382 (383–2,855)	2,139 (592–4,417)
Hypertensive disease	82,750 (36,388–124,214)	85,524 (37,607–128,377)	168,274 (73,995–252,590)

#### Costs of cardiovascular disease attributable to excessive sodium consumption to the Brazilian Health System

Tables 4 and 5 present the costs of cardiovascular diseases (CVD) to the Brazilian National Health System in 2017, considering hospitalizations and outpatient costs, respectively, which are estimated to be preventable by reducing sodium consumption to 2 g per day. Hospitalizations and outpatient costs due to cardiovascular diseases totaled US$590 million dollars in 2017, which corresponds to 1.88 billion Brazilian Reals, and 14.2% of these costs (US$84) could be prevented by reducing sodium consumption.

**Table 4:** Estimated cardiovascular disease hospitalization costs (US$ thousand) to the National Health System due to excessive sodium consumption in Brazil, 2017.

	Hospitalization costs in US$ thousands due to excessive sodium consumption (95% UI)		
Disease	Men	Women	Total
Cardiovascular disease	47,036 (19,704–74,589)	29,185 (12,255–46,132)	76,221 (32,645–118,123)
Coronary heart disease	25,616 (10,971–39,698)	12,159 (5,208–18,844)	37,775 (16,179–58,541)
Stroke	10,084 (4,307–15,545)	8,884 (3,795–13,696)	18,968 (8,124–29,395)
Heart failure	6,231 (2,661–9,825)	4,869 (2,079–7,678)	11,100 (4,754–17,203)
Aortic aneurysm	95 (40–153)	134 (57–216)	229 (98–356)
Pulmonary embolism	3,158 (998–6,281)	1,281 (405–2,548)	4,439 (1,901–6,879)
Rheumatic heart disease	542 (150–1,119)	643 (178–1,328)	1,185 (507–1,836)
Hypertensive disease	1,312 (577–1,969)	1,213 (534–1,821)	2,525 (1,081–3,913)

**Table 5:** Estimated cardiovascular disease outpatient costs to the National Health System due to excessive sodium consumption in Brazil, 2017.

	Outpatient care costs in US$ thousands due to excessive sodium consumption (95% UI)		
Disease	Men	Women	Total
Cardiovascular disease	3,225 (1,382–4,988)	2,842 (1,218–4,391)	6,067 (2,598–9,402)
Coronary heart disease	1,961 (840–3,040)	1,642 (703–2,545)	3,604 (1,543–5,585)
Stroke	754 (322–1,162)	586 (250–904)	1,340 (574–2,076)
Heart failure	104 (45–164)	90 (39–142)	194 (83–301)
Aortic aneurysm	3 (1–5)	2 (1–3)	5 (2–7)
Pulmonary embolism	12 (4–24)	17 (5–34)	29 (12–45)
Rheumatic heart disease	11 (3–23)	12 (3–24)	23 (10–35)
Hypertensive disease	380 (167–571)	492 (217–739)	873 (374–1,352)

Hospitalization costs due to excessive sodium consumption were estimated at US$76.2 million, and 87% of these costs were attributed to coronary heart disease, stroke and heart failure (Table 4). The annual outpatient costs attributable to excessive sodium consumption were estimated at US$6.1 million (Table 5). As in the case of preventable deaths, over 93% of the costs of outpatient care are related to CHD, stroke and hypertensive diseases. For both hospitalizations and outpatient care, the costs associated with men were nearly higher than those associated with women, namely, 62% of the total hospitalization and 53% of the outpatient costs.

#### Medication for high blood pressure

In Brazil, primary health care facilities freely distribute drugs for hypertension, diabetes and asthma, and these drugs are also subsidized in private pharmacies through the “*Farmacia Popular*” Program. In 2017, the Program spent US$450.5 million on anti-hypertensive drugs, and 24.7% of these costs are attributable to excessive sodium consumption among Brazilians.

In Table 6, the costs of medication for hypertension due to excessive sodium consumption reached US$109.8 million in 2017, and 56.5% of the costs were associated with women.

**Table 6:** Estimated costs of hypertension drugs (US$ thousand) to the National Health System attributed to excessive sodium consumption in Brazil, 2017.

	Costs of hypertension drugs in US$ thousand due to excessive sodium consumption (95% UI)		
Sex/age group	Men	Women	Total
30–49 years	9,397 (4,025–14,562)	12,250 (5,247–18,985)	21,647 (9,271–33,547)
50 to 64 years	20,013 (8,571–31,015)	26,418 (11,315–40,942)	46,431 (19,886–71,956)
Over 64 years	18,190 (7,791–28,191)	23,548 (10,085–36,493)	41,738 (17,876–64,683)
Total	47,600 (20,387–73,767)	62,216 (26,647–96,419)	109,816 (47,034–170,187)

#### Productivity costs due to premature death by CVD

In 2017, as shown in Table 7, productivity losses due to premature deaths attributable to excessive sodium consumption reached US$752.7 million. We found that 72.3% (US$544.6 million) of the productivity losses among men were due to a higher sodium consumption, combined with higher death rates by CVDs and the pension age (currently, in Brazil, the pension age is 60 years of age for women and 64 years for men).

**Table 7:** Estimated productivity losses (US$ thousand) of premature CVD deaths due to excessive sodium consumption in Brazil, 2017.

	Productivity losses in US$ million (95% UI)		
Sex/age group	Men	Women	Total
30–34 years	45,730 (19,749–69,837)	21,841 (9,432–33,354)	67,570 (29,182–103,191)
35–39 years	71,431 (30,849–109,087)	35,759 (15,443–54,609)	107,190 (46,292–163,696)
40–44 years	90,601 (39,128–138,362)	43,816 (18,923–66,914)	134,416 (58,051–205,276)
45–49 years	109,911 (47,468–167,853)	48,641 (21,007–74,283)	158,552 (68,475–242,136)
50–54 years	122,265 (52,803–186,719)	40,024 (17,285–61,123)	162,289 (70,088–247,842)
55–59 years	100,585 (43,440–153,610)	18,008 (7,777–27,501)	118,593 (51,217–181,111)
60–64 years	4,121 (1,780–6,293)	0 (0–0)	4,121 (1,780–6,293)
Total	544,644 (235,217–831,761)	208,088 (89,868–317,784)	752,732 (325,085–1,149,545)

Productivity losses due to premature death increase with age and peak at 50 to 54 years among men and at 45 to 49 years among women. Over 62% of the productivity losses for both sexes were concentrated within the 30 to 50 years of age range, which represents a large burden to the economy, considering the impact on the labor force of the country.

## Discussion

Excessive sodium consumption leads to high blood pressure, which increases the risk of cardiovascular diseases, such as coronary heart disease, stroke, heart failure, pulmonary embolism, aortic aneurysm, rheumatic heart disease, and hypertensive disease [26][25]. The populations of most countries around the world, including Latin America, consume much over 2 g [35][36][37] of sodium per day, and the health and economic impacts of interventions are very important considerations for policymakers.

This study uses an original macrosimulation approach to link sodium consumption with CVDs mediated by high blood pressure and shows the impact of sodium consumption on deaths and the costs to the health system and society in Brazil. The results confirm that Brazil follows the estimates, provided by the GBD 2017 study, of the burden of sodium consumption, as a major risk factor for death and disease. Excessive sodium consumption by Brazilians was estimated to be responsible for 6.4% of all preventable deaths and 15.0% of CVD deaths in 2017, which corresponds to 47,017 estimated deaths.

Excessive sodium consumption was also estimated to be responsible for US$76.2 million, in terms of the total hospitalization costs, and US$6.1 million in outpatient care costs to the Brazilian National Health System in 2017. Because outpatient costs do not account for primary health care, which covers over 70% of the population and is responsible for the first level of health care, especially to people with chronic diseases, the attributable costs of CVDs caused by excessive sodium consumption, presented here, are likely underestimated.

The costs of hypertension drugs corresponded to over US$450 million (60% of the costs of the publicly distributed and subsidized *Farmacia Basica* Program) in 2017, and almost one fourth of these costs could be averted by reducing sodium consumption to recommended levels (US$109.8 million). While the costs of hypertension drugs have increased, since the creation of the program in 2004, the free or subsidized supply of hypertension drugs has proved to be an effective secondary CVD prevention strategy. As a result, the expansion of access to medicines for the treatment of chronic diseases, such as hypertension, has resulted in an average annual reduction of 27.6% in hospitalizations and 8.0% in deaths by cardiovascular disease in Brazil [38].

Regarding age differences, the burden of excessive sodium consumption generally increases with age, although a significant number of attributable deaths and costs already arise in younger groups. This can be explained by the higher sodium consumption among adolescents and adults and by the higher relative risks associated with CVDs in younger age groups. In terms of gender differences, women tend to access health services more frequently and consume less sodium than men. As a result, the burden of sodium-related deaths is higher in men, while the health system and societal costs tend to be similar between women and men.

Health economics methodologies also allow estimations related to dietary risks and NCDs, such as sodium consumption, including the years of life lost, years of productive life lost and the burden of premature deaths by CVD, to be made. The indirect societal costs are not limited to these analyses and may include costs of premature retirements, presentism, absenteeism and other costs of diseases to families and communities.

Non-communicable diseases were estimated to have caused losses of up to 7.3% of the Brazilian Gross Domestic Product (2.2% in early retirement and 5.1% in absenteeism and presentism) in 2015 [39]. In the US, hypertension, coronary heart disease and stroke cause a productivity loss of 2.8% due to absenteeism and a 6.8% loss due to presentism per employee, per year [40].

### Study strengths and limitations

The strengths of this study include the use of publicly available, nationally representative and robust data from Brazilian administrative health information systems concerning the deaths and costs to the health system, disaggregated by sex, education, race and age group over time. The estimations are also based on data from national population surveys on food consumption, labor force characteristics and national life table estimates. These macrosimulation approaches are based on GBD methodologies, so intra- and inter-country and regional comparisons are possible under similar conditions relating to the relative risks associated with CVDs. Finally, estimations can be reproduced for specific years or periods of years from data that are normally available from most countries and international organizations.

In general, the results of the modeling studies using probabilistic approaches represent an estimate of the magnitude of the impact in different policy or counterfactual scenarios and must be interpreted as so. The uncertainty intervals of the results incorporate the uncertainty of many of its inputs, including the uncertainty of risk factor exposure (as the variance in sodium intake) and relative risks from meta-analyses, through the sensitivity analysis.

The macrosimulation approaches for attributable health and cost burden of excessive sodium intake have several limitations. Firstly, these cross-sectional NCD scenario models are incapable of incorporating the effect of time lag between exposure and disease outcome. Also, the relative risk estimates used to parameterize the models are based on published meta-analyses which may be not be the most current in the scientific literature now. Regarding the use of data in the models, these macrosimulations do not account for all sources of data heterogeneity and allow the risk for cardiovascular disease to be conditional on age, sex and exposure to risk factors (sodium intake).

The main limitation of the cost component of this study is the underestimation of the costs, which is due to the multiple direct and indirect costs of NCDs and the impossibility of calculating the actual *per capita* costs of the diseases. The analyses also do not account for the costs of hospitalizations and other health costs, such as health insurance and out-of-pocket costs, as well as the costs of hypertension and CVDs to primary health care. Besides, the macrosimulation approaches used in the study are static, so the analyses are limited to the comparison of baseline and counterfactual scenarios and projections, such as DALYs and QALYs.

The attributable costs estimates may also be underestimated because of differences between the studies on relative risks used by PRIME and larger and more recent meta-analyses [41][42], which consider more age-specific differences, especially regarding adolescents and young adults. Methodologically, we chose to use the original PRIME references [25][26] in order to harmonize the attributable deaths estimated by PRIME with the cost-of-illness estimates in this study, producing internally comparable and conservative cost estimates.

Another limitation of this study is that the data concerning sodium consumption in Brazil was estimated using spot urine from a national survey held in 2013, which has proven to be relatively accurate for sodium consumption close to the average intake, but that may bias estimates at both extremes of intake distribution [43]. The use of more recent and accurate data (24h urine) would provide an indication of whether the sodium consumption remains stable or has changed.

## Conclusions

This study conservatively estimates that yearly 47,017 deaths, over US$84 million (268 million Brazilian Reals) in costs to the Brazilian Health System and US$827 million in costs of premature deaths to the country’s economy could be prevented or avoided, if the population consumed an average of 2 g of sodium (5 g of salt) per day. The methodologies presented in this study may be used to estimate the potential impact of different health strategies on sodium reduction and select “best buy” interventions for policy-makers.

Our findings suggest that sodium reduction policies in Brazil must be strengthened in order to reduce the significant burden of excessive sodium consumption on the country. These policies might include health promotion, food regulation, nutritional counseling, prevention, surveillance and disease treatment, which must be prioritized according to their cost-effectiveness.

Further research using health and economic modeling approaches is needed, especially in developing countries, to monitor health expenditure and to aid in improving policy implementation and the allocation of public funds in order to reduce preventable deaths by cardiovascular diseases and other NCDs and their toll on health systems and society.

## Supporting information

S1 File (DOCX)
