# Supplementary material for: Modelling the effect of compliance with WHO salt recommendations on cardiovascular disease mortality and costs in Brazil
Source: PLoS One. 2020 Jul 9;15(7):e0235514. doi: 10.1371/journal.pone.0235514 (PMC7347203; doi:10.1371/journal.pone.0235514)
Supplement: S1 File — (DOCX) [file pone.0235514.s001.docx]

**Supplemental Material**

Supplement to Nilson. EAF; Metlzer. AB. Labonté. ME. Jaime. PC. Modelling the Effect of Compliance with WHO Salt Recommendations on Cardiovascular Disease Mortality and Costs in Brazil. 2020. (pre print version)

Section S1. Additional methodological details

Tables

.

Section S1. Additional methodological details

Materials and methods

Salt consumption estimates

The most recent nationally representative salt consumption data was assessed from spot urine collected in 2013, as part of the Brazilian National Health Survey (NHS), a household survey with a representative sample of the Brazilian adult population and planned a subsample of 25% of the adults interviewed for biochemical laboratory exams (approximately 12,000 individuals). At the end of field collection, the intended subsample was not reached, and 8,952 spot urine samples were collected. Additionally, 10% of the urine samples were lost because of incomplete tests or insufficiency of material. As a result, 8,083 urine samples were analyzed. Due to the sample losses for the laboratory exams, researchers adopted a post-stratification method, which included variables such as gender, age, ethnicity, and region, aiming at reducing non-representativeness bias, and re-estimating sample weights.(1)

The 24-hour urinary salt excretion was estimated by the equation of Tanaka, which was validated previously for the Brazilian population in a substudy in the city of Vitória, comparing spot and 24 hour urine samples and using creatinine excretion for predicted for 24 hour (CrPr24h), based on age, weight, and height of the individuals. According to this validation study, the spot urine analysis adequately estimated the average population intake, although the Tanaka formula tended to underestimate salt intake in higher salt intake ranges (2).

Due to the final sample size of urine samples and to the limitations of the Tanaka method, the estimated salt intake was described according to gender (male and female), age group (18 to 29; 30 to 44; 45 to 59; and 60 or older), schooling (illiterate up to incomplete elementary school; complete elementary school up to incomplete high school; and complete high school and higher), ethnicity (white, black, multiracial, and other), and region of Brazil (North, Northeast, Midwest, Southeast, and South). (3)

Mortality data

The Mortality Information System (SIM) is managed by the Ministry of Health of Brazil and provides recent and historical microdata and public aggregated tables for information on deaths in the country.

The Mortality Information System (SIM) supports the collection, storage and management process of death registries, in Brazil, which is mandatory in all cities. Mortality data are periodically sent to the State Secretaries of Health and then to the Ministry of Health. The historical data stored by the system produce indicators that subsidize managers through managerial consultations through situational dashboard health consultation apps in consolidated databases (4)

The public data provided for SIM (5) include disaggregation by place and year of death, chapter, group, category and cause of death according to ICD10, sex, age, location, race, education and marital status of the deceased.

National Health System Costs

Cost-of-disease analyses normally require direct and indirect costs of morbidity which can be identified by the International Code of Disease for more precise estimations.

For example, the Brazilian National Health System (SUS – Sistema Único de Saúde) has many open databases with identification of ICD codes, sex and age group, as the SUS Outpatient Information System (Sistema de Informação Ambulatorial, SIA/SUS) and Hospital Information System (Sistema de Informação Hospitalar, SIH/SUS). The “Farmácia Popular” Program (public hypertension, diabetes, and asthma drug provision program)5 also provides information on the costs of hypertension drugs and the National Supplementary Health Agency provides publicly available information on the refunds to SUS by private health plans and insurances.

The official costs of the treatment of disease to the Brazilian National Health System (SUS) are consolidated by the Hospital Information System – SIH/SUS (6) and the Ambulatory Information System – SIA/SUS (7), which are sent to the Ministry of Health by all public and private health care facilities that work for SUS.

The consolidated data on hospitalizations includes location, costs, duration and cause (ICD 10) of the hospitalizations and patient information, such as sex, age and race, disaggregated by month and year. The data on the costs of outpatient (ambulatory) care is also consolidated by the Ministry of Health and tables with data on location, disease and patient characteristics are publicly available for visualization and download.

**CVD MORTALITY MODULE**

**Modeling of prevented or postponed deaths**

**PRIME model and estimation of the effect of reduction of salt intake on the mortality from cardiovascular diseases.**

The Preventable Risk Integrated ModEl (PRIME) is a macro-simulation model which was developed to estimate the impact of changes in the distribution of NCD risk and protection factors, including dietary variables, on mortality from chronic diseases. The complete PRIME model uses inputs such as dietary consumption (including alcohol consumption) and nutrient intakes, physical activity, height, BMI, and smoking status.

The observed salt intake from the Brazilian National Health Survey of 2013 was used to determine the reference (baseline) scenario and the counterfactual scenario considered that average salt consumption was reduced to 5g/day, introduced in the PRIME model.

***Mortality and demographic data***

The cardiovascular diseases related to excessive salt intake used in the model include coronary heart disease, stroke, hypertensive disease, heart failure, aortic aneurysm, pulmonary embolism, and rheumatic heart disease (ICD-10: I05-15, I20-26, I50, I60-69, I71). Mortality data for each disease was obtained from publicly available databases of the National Health information System on Deaths (SIM – *Sistema de Informações de Mortalidade*), stratified by sex and five-year age groups and ICD-10 codes (5).

The age and sex structure of the population for the same year was determined using publicly available data from the Brazilian Institute of Geography and Statistics (IBGE) for 2017, also stratified by sex and five-year age groups (8).

All mortality and demographic data are publicly available by the Ministry of Health (5) and by the Brazilian Institute of Geography and Statistics (8).

***Baseline and counterfactual scenario of dietary distribution***

The dietary intakes of salt from the 2013 National Health Survey (NHS) were used as the baseline ‘reference’ for analysis. Then, the counterfactual scenario considered an average salt consumption of 2g/day for the modelling of averted or postponed deaths, using the Preventable Risk Integrated ModEl (PRIME) (9). We assumed salt intake did not change from 2013 and 2017 and that the standard deviation (SD) of salt intake in the counterfactual scenario was proportional to the SD at the baseline.

***Parameterization of the association between dietary consumptions and chronic diseases***

The PRIME model estimates death rates associated to chronic diseases in a given situation using relative risks of different levels of nutrient intakes on chronic diseases and the baseline distribution. In the case of salt intake, the model has a two-step approach: first, it simulates the impact of changes in salt intake on blood pressure, and then it simulates the impact of the changes in blood pressure on the number of deaths averted or delayed from cardiovascular diseases. The model uses a log-normal distribution of salt intake in the population, for both the baseline and counterfactual scenarios, for sex and 5-year age groups, using the mean and standard deviation of salt intake and the population data. The net impact of the changes in the risk factor is represented by the difference of the averted deaths number between the baseline and counterfactual scenarios (9).

***Uncertainty analyses***

Considering the uncertainty of outcomes in the model, performing a probabilistic sensitivity analysis is recommended in order to explore the potential effects of reducing salt consumption on the risk factors for CVDs. In this paper, simulations were performed using the Monte Carlo methodology, which allows a stochastic (random) variation of parameters based on the sizes of the effects obtained from the literature. By using this technique, the model results were recalculated iteratively and uncertainty intervals of 95% (UI 95%) were generated for the median using the bootstrap percentile method. The model simulation was implemented the Monte Carlo analysis embedded in the PRIME and running 10,000 iterations (draws) from specified probabilistic distributions for the model input variables (salt intake, deaths and relative risks).

The macrosimulation models (both PRIME and cost evaluation) implement a Monte Carlo approach to estimate uncertainty intervals (UI) for each scenario (KOERKAMP et al., 2011). Each simulation runs 10,000 times. For each iteration, log-normal distributions of salt consumption, together with the relative risks from literature, are assumed for the input parameters.

The macrosimulation framework does not allow stochastic uncertainty, such as microsimulations (patient-level models), nevertheless parameter uncertainty and individual heterogeneity in our study are reflected in the reported UI. Modeling patient heterogeneity allows analyses based on individual patient characteristics that can influence the outcomes of a decision model. In this study, we have modelled discrete subgroups to represent patient heterogeneity, considering gender or age ranges, within which all individuals are assumed identical. The subgroup-specific characteristics result in subgroup-specific expected outcomes and the discrete distribution of the expected outcome across all subgroups reflects the patient heterogeneity. In addition, parameter uncertainty (2nd order uncertainty) expresses the results from lack of perfect knowledge on their true values, so parameters as relative risks are typically represented by a probability distribution that can be propagated through the model using Monte Carlo simulation, resulting in a distribution of the expected outcome, reflecting lack of perfect knowledge.

Therefore, we allow the risk for CHD to be conditional on individual characteristics (i.e. age, sex, exposure to risk factors – sodium intake) and consider the estimate the uncertainty of the relative risks due to sampling errors through the Monte Carlo analyses.

The structure of the models is grounded on fundamental epidemiological ideas and well-established causal pathways; therefore, we considered this type of uncertainty relatively small and did not study it.

Parameter estimation and uncertainty follow the Modeling Good Research Practices (BRIGGS et al., 2012), for both PRIME and the attributable cost estimation model, by incorporating different concepts related to uncertainty, including the stochastic (first-order) uncertainty, the parameter (second-order) uncertainty, the structural uncertainty and the heterogeneity.

The framework of these macrosimulations allows stochastic uncertainty, parameter uncertainty, and individual heterogeneity to be reflected in the reported UI. In this kind of modelling, the heterogeneity encompasses the variability between patients that can be attributed to their characteristics, which in regression terms, would correspond to beta coefficients or the extent to which dependent variable varies by patient characteristics. In the case of these models, which are based on the sodium-hypertension-cardiovascular outcome rationale, the four sources of uncertainty are incorporated in the model, through Monte Carlo analysis, parameter parametrization and assumptions of the decision model (as the log-linear regression for the distribution of salt consumption). As macrosimulations, the heterogeneity is not considered at the individual level (as for microsimulations) but is assessed though the different exposures (sodium intake) and the parametrized relative risks that are specific to each exposure level, age-group and sex (Tables 8, 9 and 10A to 10D), in order to allow the reproducibility of the results.

The uncertainties, therefor, were incorporated in the final UI by implementing a 2nd order Monte Carlo analysis to estimate uncertainty in each scenario (KOERKAMP et al., 2011). The 2nd order Monte Carlo analysis uses two loops of iterations: the inner loop represents the variability (as the SD of the exposures) and the outer loop represents parameter uncertainty (as the RRs used in the parametrization). This also allows the model to incorporate the usual random error (sampling error) in the RR and exposure prevalence as well as other potential sources of uncertainty such as uncontrolled confounding or extrapolation from a source to a target population, because of the assumption of the portability of the RRs from the metanalyses. In case of the modeling uses in this study, the final population attributable fractions (PAF) are based on the weighted sum of the PAF for each exposure, sex and age-group strata. Again, after repeated draws and repeated calculations of the AF, Monte Carlo limits can be obtained. Patient heterogeneity is represented by frequency distributions and analyzed with Monte Carlo simulation. Parameter uncertainty is represented by probability distributions and analyzed with 2nd-order Monte Carlo simulation (aka probabilistic sensitivity analysis). (STEENLAND; ARMSTRONG, 2006).

**HEALTH ECONOMICS ANALYSIS**

**Modeling the direct and indirect costs of CVDs associated to hypertension in the Brazilian National Health System**

**The cost-of-illness methodology adapted to a dietary risk factor (salt intake).**

The cost-of-illness model is a macrosimulation model, which was developed and validated to link the impact of changes in the distribution of salt intake to the costs of cardiovascular disease. The model’s inputs are the dietary salt intake, relative risks of salt as a risk factor for hypertension, and of hypertension as a risk factor for cardiovascular diseases. Figure 1 shows a simplified model structure and a full description of the cost-of-illness model, including all the parameters used, has been described in depth by its developers elsewhere (10).

The observed salt intake from the Brazilian National Health Survey of 2013 was used to determine the reference (baseline) scenario and the counterfactual scenario considered that the average salt consumption among adults was reduced to 5g/day.

Figure 1. Steps in the analysis of the costs of cardiovascular diseases attributable to salt consumption.


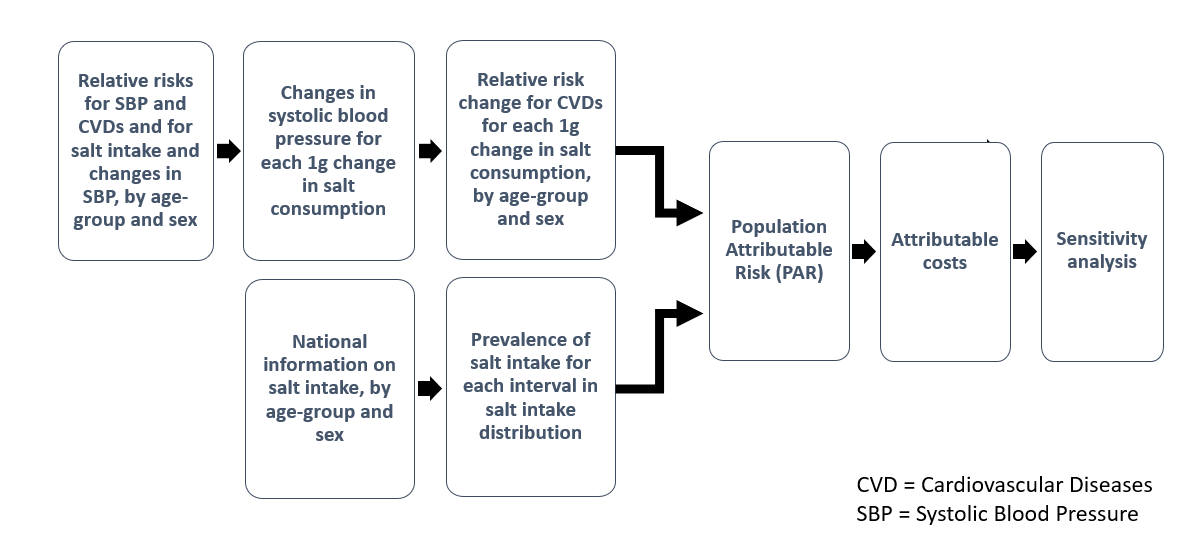


***Data on the direct costs of CVDs to the Nation al Health System***

The cardiovascular diseases related to excessive salt intake used in the model include coronary heart disease, stroke, hypertensive disease, heart failure, aortic aneurysm, pulmonary embolism, and rheumatic heart disease (ICD-10: I05-15, I20-26, I50, I60-69, I71). Data on hospitalization costs and outpatient costs in the Brazilian National Health System were obtained from publicly available databases of the National Health information Systems on Deaths (SIH – *Sistema de Informações Hospitalares* and SIA *– Sistema de Informações Ambulatoriais*), stratified by sex and age groups (7)(6)(5) and with costs identified by cause (ICD-10).

***Baseline and counterfactual scenario of dietary distribution***

The dietary intakes of salt from the 2013 NHS were used as the baseline ‘reference’ for analysis. Then, the counterfactual scenario considered an average intake consumption of 2g/day for the modelling of attributable costs, using the adaptation of the cost-of illness methodology, respectively (11). We assumed salt intake did not change from 2013 and 2017 and that the standard deviation (SD) of salt intake in the counterfactual scenario was proportional to the SD at the baseline.

Due to the final sample size of spot urine samples and to the limitations of the Tanaka method, the distributions of salt intake used in the model considered a stratification by sex and by age group (18 to 29y; 30 to 44y; 45 to 59y; and 60y or older). (3)

***Parameterization of the association between dietary consumptions and chronic diseases***

The cost-of-illness methodology uses the same set of relative risks for salt/sodium intake, hypertension and CVDs part as the PRIME model to estimate costs of cardiovascular outcomes mediated by hypertension that are attributable to salt intake, comparing counterfactual scenarios to a baseline intake. Therefore, the results on deaths and costs can complement each other regarding the analysis of the burden of excessive salt intake. The model has a two-step approach: first, it parametrizes the impact of changes in salt intake on blood pressure, and then it parametrizes the impact of the changes in blood pressure on the cardiovascular diseases.

**Cost-of-illness estimation**

The indirect costs of excessive salt consumption were estimated using the human capital approach to the attributable costs to premature deaths. The Human Capital Approach is the traditional method to calculating lost productivity (12), and calculates the present value of potential time in the workforce (the measure of productivity) using market wages (13). In the case of the costs of premature deaths, we used the results from the prevented or postponed deaths generated by PRIME to calculate the total YPLL (Years of Productive Life Lost), ie, for each age and sex group, the total years from the age of death to retirement age multiplied by the number of premature deaths (14). Finally, the productivity losses in terms of costs to society are calculated by multiplying the YPLL by the employment rate and the average wage of the economically active population in 2017 (15) – Tables 19 and 20.

A full description of the cost-of-illness, including all the parameters and methodological steps of the model, can be found in Nilson et al, 2020 (10) and the detailed steps of the parametrization of the relationship between sodium intake and systolic blood pressure and, then, of systolic blood pressure and cardiovascular outcomes are:

*Calculating the changes in systolic blood pressure (SBP) for each gram of salt added to the diet*

The impact of changes in salt consumption on blood pressure is based on a meta-analysis of random controlled trials that estimated that a 6g of salt per day reduction would result in a 5.8 mmHg reduction in SBP, after a 4-week intervention period (16).

In the model, salt consumption is considered a continuous risk factor, which is directly associated with a specific health outcome (blood pressure). Thus, it is possible to parametrize the results as a linear association in intervals of consumption so that, for each gram reduction of salt from the diet, SBP will be reduced by 5.8 mmHg/6g = 0.933 mmHg.

SBP change is then estimated for the midpoint of each interval in the distribution of salt intake, using the following formula:

**ΔSBP = 0.933 x S**

Where

ΔSBP = change in the systolic blood pressure

S = midpoint of the salt intake interval

We have divided salt intakes into four intervals (<5g, 5 to 8g, 8 to 12g, and over 12g). So, in this analysis, the corresponding midpoints or reference points for estimating the prevalence of salt intake among adults for each interval of the distribution were 5g, 6.5g, 10g and 14g/day.

*Calculating the relative risk by disease for each gram of salt added to the diet*

After estimating the expected changes in SBP from changing salt consumption, the next step of the costing tool calculating the differential relative risk associated with the increase/decrease of SBP for each disease, considering age and sex groups, for each interval of salt intake. These relative risks can be calculated for coronary heart disease, stroke, hypertensive disease, heart failure, pulmonary embolism, rheumatic heart disease, and aortic aneurysm (17).

The estimates of relative risks according to age, sex, and interval of salt intake distribution for each cardiovascular outcome are based on the following formula (detailed in Tables 10A, 10B, 10C and 10D):

**RRi = RR ^(x-y)/u**

Where:

RR = relative risk for the sex/age group (meta-analysis)

X = midpoint of the salt intake interval

Y = midpoint of the first salt consumption interval (equivalent to consuming less than 5g/day, therefore with a relative risk of 1)

U= unit of change in SBP described in the scientific literature (20 mmHg)

*Calculating the population attributable risk for each 1g interval of salt intake*

Firstly, the prevalence of salt intake for each interval in the distribution must be estimated, according to sex and age groups. The World Health Organization recommends consuming less than 5 g/day of salt for adults, so the intake distribution in the modeling starts at 5 g/day of salt and the other salt intake intervals are added (1 g or other) until the upper limit of intake in the salt intake scenario under analysis.

Then, the Population Attributable Risk (PAR) is calculated for each interval of salt intake, considering the prevalence of salt intake for each interval and its specific relative risk (as calculated in the third step of the methodology). As a result, PAR estimates the proportion of cases of the specific outcome that is associated with the risk factor (salt intake), as shown in the following formula and detailed on tables 11 to 16.

**PARi=P(RRi−1)/[P(RRi−1)+1]**

Where:

P = Prevalence of salt intake in the interval and strata (age and sex group), and

RRi = Relative risk for each 1g interval of salt to the CVD outcome.

*Calculating the attributable costs*

The attributable cost for salt intake interval and strata (age and sex group) is estimated by multiplying the costs in each stratum (age and sex group) by its PAR. Finally, total attributable costs are the sum of the attributable costs for all strata:

**Ct = ∑(PARi * Ci)**

Where:

PARi = population attributable risk for each interval of salt intake distribution

Ci= associated cost for each cardiovascular outcome for each and strata (age and sex group)

The total attributable costs for hospitalization costs are detailed in tables 17 and 18.

***Uncertainty analyses***

Considering the uncertainty of outcomes in the model, performing a probabilistic sensitivity analysis is recommended in order to explore the potential effects of reducing salt consumption on the risk factors for CVDs. In this paper, simulations were performed using the Monte Carlo methodology, which allows a stochastic (random) variation of parameters based on the sizes of the effects obtained from the literature. By using this technique, the model results were recalculated iteratively and uncertainty intervals of 95% (UI 95%) were generated for the median using the bootstrap percentile method.

This study used the Ersatz package (18) and MS Excel for a stochastic variation of the parameters of the cost-of-illness model, based on the sizes of the effects obtained from the literature. So, starting from the Pert standard distribution function for salt intake, costs and relative risks, the uncertainty intervals (UI) for the medians were generated using the bootstrap percentile method, running 10,000 iterations.

The macrosimulation models (both PRIME and cost evaluation) implement a Monte Carlo approach to estimate uncertainty intervals (UI) for each scenario (KOERKAMP et al., 2011). Each simulation runs 10000 times. For each iteration, log-normal distributions are assumed for the input parameters.

The macrosimulation framework does not allow stochastic uncertainty, such as microsimulations (patient-level models), nevertheless parameter uncertainty and individual heterogeneity in our study are reflected in the reported UI. Modeling patient heterogeneity allows analyses based on individual patient characteristics that can influence the outcomes of a decision model. In this study, we have modelled discrete subgroups to represent patient heterogeneity, considering gender or age ranges, within which all individuals are assumed identical. The subgroup-specific characteristics result in subgroup-specific expected outcomes and the discrete distribution of the expected outcome across all subgroups reflects the patient heterogeneity. In addition, parameter uncertainty (2nd order uncertainty) expresses the results from lack of perfect knowledge on their true values, so parameters as relative risks are typically represented by a probability distribution that can be propagated through the model using Monte Carlo simulation, resulting in a distribution of the expected outcome, reflecting lack of perfect knowledge.

Therefore, we allow the risk for CHD to be conditional on individual characteristics (i.e. age, sex, exposure to risk factors – sodium intake) and consider the estimate the uncertainty of the relative risks due to sampling errors through the Monte Carlo analyses.

Parameter estimation and uncertainty follow the Modeling Good Research Practices (BRIGGS et al., 2012), for both PRIME and the attributable cost estimation model, by incorporating different concepts related to uncertainty, including the stochastic (first-order) uncertainty, the parameter (second-order) uncertainty, the structural uncertainty and the heterogeneity.

The framework of these macrosimulations allows stochastic uncertainty, parameter uncertainty, and individual heterogeneity to be reflected in the reported UI. In this kind of modelling, the heterogeneity encompasses the variability between patients that can be attributed to their characteristics, which in regression terms, would correspond to beta coefficients or the extent to which dependent variable varies by patient characteristics. In the case of these models, which are based on the sodium-hypertension-cardiovascular outcome rationale, the four sources of uncertainty are incorporated in the model, through Monte Carlo analysis, parameter parametrization and assumptions of the decision model (as the log-linear regression for the distribution of salt consumption). As macrosimulations, the heterogeneity is not considered at the individual level (as for microsimulations) but is assessed though the different exposures (sodium intake) and the parametrized relative risks that are specific to each exposure level, age-group and sex (Tables 8, 9 and 10A to 10D), in order to allow the reproducibility of the results.

The uncertainties, therefor, were incorporated in the final UI by implementing a 2nd order Monte Carlo analysis to estimate uncertainty in each scenario (KOERKAMP et al., 2011). The 2nd order Monte Carlo analysis uses two loops of iterations: the inner loop represents the variability (as the SD of the exposures) and the outer loop represents parameter uncertainty (as the RRs used in the parametrization). This also allows the model to incorporate the usual random error (sampling error) in the RR and exposure prevalence as well as other potential sources of uncertainty such as uncontrolled confounding or extrapolation from a source to a target population, because of the assumption of the portability of the RRs from the metanalyses. In case of the modeling uses in this study, the final population attributable fractions (PAF) are based on the weighted sum of the PAF for each exposure, sex and age-group strata. Again, after repeated draws and repeated calculations of the AF, Monte Carlo limits can be obtained. Patient heterogeneity is represented by frequency distributions and analyzed with Monte Carlo simulation. Parameter uncertainty is represented by probability distributions and analyzed with 2nd-order Monte Carlo simulation (aka probabilistic sensitivity analysis). (STEENLAND; ARMSTRONG, 2006).

The structure of the models is grounded on fundamental epidemiological ideas and well-established causal pathways; therefore, we considered this type of uncertainty relatively small and did not study it.

References

1. Szwarcwald CL, Malta DC, Almeida PRB de S, Damacena W da S, Nogueira G, Pereira CA, et al. Laboratory exams of the National Health Survey: methodology of sampling, data collection and analysis. Rev Bras Epidemiol [Internet]. 2019;22(Supp.2):E190004. Available from: https://doi.org/10.1590/1980-549720190004.supl.2

2. Mill JG, Rodrigues SL, Baldo MP, Malta DC, Szwarcwald CL. Estudo de validação das equações de tanaka e de kawasaki para estimar a excreção diária de sódio através da coleta da urina casual. Rev Bras Epidemiol [Internet]. 2015;18:224–37. Available from: http://www.scielo.br/pdf/rbepid/v18s2/1980-5497-rbepid-18-s2-00224.pdf

3. Mill JG, Malta DC, Machado ÍE, Pate A, Pereira CA, Jaime PC, et al. Estimativa do consumo de sal pela população brasileira: resultado da Pesquisa Nacional de Saúde 2013. Rev Bras Epidemiol [Internet]. 2019;22(suppl 2):E190009. Available from: http://www.scielo.br/pdf/rbepid/v22s2/1980-5497-rbepid-22-s2-e190009-supl-2.pdf

4. Morais RM de, Costa AL. An evaluation of the Brazilian Mortality Information System. Saúde em Debate [Internet]. 2017;41(N Especial):101–17. Available from: https://doi.org/10.1590/0103-11042017s09

5. Ministério da Saúde. SIM - Mortality Information System [Internet]. 2017. Available from: http://tabnet.datasus.gov.br/cgi/deftohtm.exe?sim/cnv/obt10uf.def

6. Ministério da Saúde. SIH-SUS - Hospital Information System [Internet]. 2017. Available from: http://tabnet.datasus.gov.br/cgi/deftohtm.exe?sih/cnv/niuf.def

7. Ministério da Saúde. SIA-SUS - Ambulatory Information System [Internet]. 2017. Available from: http://www2.datasus.gov.br/DATASUS/index.php?area=0901&item=1&acao=22&pad=31655

8. IBGE. Brazilian Population Estimates [Internet]. 2017. Available from: https://www.ibge.gov.br/en/statistics/social/population/18448-population-estimates.html?=&t=o-que-e

9. Scarborough P, Harrington RA, Mizdrak A, Zhou LM, Doherty A. The Preventable Risk Integrated ModEl and Its Use to Estimate the Health Impact of Public Health Policy Scenarios. Scientifica (Cairo). 2014;2014:748750.

10. Nilson EAF, Silva EN da, Jaime PC. Developing and applying a costing tool for hypertension and related cardiovascular disease: attributable costs to salt/sodium consumption. J Clin Hypertens [Internet]. 2020;00:1–7. Available from: https://doi.org/10.1111/jch.13836

11. Oliveira ML De, Santos LMP, Silva EN da. Bases metodológicas para estudos de custos da doença no Brasil. Rev Nutr [Internet]. 2014;27(5):585–95. Available from: http://www.scielo.br/pdf/rn/v27n5/1415-5273-rn-27-05-00585.pdf

12. Łyszczarz B, Nojszewska E. Productivity losses and public finance burden attributable to breast cancer in Poland, 2010-2014. BMC Cancer [Internet]. 2017;17(1):676. Available from: https://doi.org/10.1186/s12885-017-3669-7

13. Zhang W, Bansback N, Anis AH. Measuring and valuing productivity loss due to poor health: A critical review. Soc Sci Med [Internet]. 2011;72(2):185–92. Available from: https://doi.org/10.1016/j.socscimed.2010.10.026

14. Pearce A, Hanly P, Sharp L, Soerjomataram I. The Burden of Cancer in Emerging Economies: Productivity Loss as an Alternative Perspective. Value Heal [Internet]. 2015;18(7):A336. Available from: https://www.valueinhealthjournal.com/article/S1098-3015(15)02194-4/pdf

15. IBGE. Continuous National Household Sample Survey - Continuous PNAD [Internet]. 2017. Available from: https://www.ibge.gov.br/en/statistics/full-list-statistics/16809-quarterly-dissemination-pnad2.html?edicao=22227&t=o-que-e

16. He FJ, Li J, MacGregor GA. Effect of longer term modest salt reduction on blood pressure: Cochrane systematic review and meta-analysis of randomised trials. BMJ [Internet]. 2013;346:f1325. Available from: https://doi.org/10.1136/bmj.f1325

17. Lewington S, Clarke R, Qizilbash N, Peto R, Collins R, Lewington S, Clarke R, Qizilbash N, Collins R PR. Age-specific relevance of blood pressure to vascular disease in one million people in 61 cohort studies. Prospective Studies Collaboration. Lancet [Internet]. 2002;60(9349):1903–13. Available from: https://doi.org/10.1016/s0140-6736(02)11911-8

18. Barendregt JJ. Ersatz User Guide [Internet]. 2017. 76 p. Available from: http://www.epigear.com/index_files/Ersatz User Guide.pdf

19. Lewington S, Clarke R, Qizilbash N, Peto R, Collins R. Age-specific relevance of usual blood pressure to vascular mortality: A meta-analysis of individual data for one million adults in 61 prospective studies. Lancet. 2002;360(9349):1903–13.

**Supplementary Tables**

This appendix has been provided by the authors to give additional information about their work.

**Table 1. Brazilian population data. 2017 (Brazilian Institute of Geography and Statistics – IBGE)**

| **Age** | **Male** | **Female** |
| --- | --- | --- |
| **15-19y** | 8,710,079 | 8,430,049 |
| **20-24y** | 8,621,967 | 8,434,408 |
| **25-29y** | 8,634,062 | 8,542,758 |
| **30-34y** | 8,816,350 | 8,821,067 |
| **35-39y** | 7,879,629 | 7,976,620 |
| **40-44y** | 6,882,229 | 7,062,001 |
| **45-49y** | 6,266,088 | 6,536,311 |
| **50-54y** | 5,659,602 | 6,027,703 |
| **55-59y** | 4,678,702 | 5,120,890 |
| **60-64y** | 3,655,025 | 4,141,984 |
| **65-69y** | 2,672,043 | 3,172,657 |
| **70-74y** | 1,793,543 | 2,283,021 |
| **75-79y** | 1,222,286 | 1,691,282 |
| **80-84y** | 623,388 | 1,031,099 |
| **85y+** | 623,388 | 1,031,099 |

**Table 2. Mortality from Cardiovascular Diseases in Brazil, 2017 (National Mortality Information System – SIM)**

|  | **I60-I69: Cerebrovascular diseases** | **I20-I25: Ischaemic heart diseases** | **I10-I15: Hypertensive disease** | **I50: Heart failure** | **I71: Aortic aneurysm** | **I26: Pulmonary embolism** | **I05-09: Rheumatic heart disease** | **Total** |
| --- | --- | --- | --- | --- | --- | --- | --- | --- |
| **M30-34y** | 301 | 517 | 128 | 64 | 43 | 59 | 25 | 1.137 |
| **M35-39y** | 536 | 955 | 250 | 128 | 83 | 99 | 30 | 2.081 |
| **M40-44y** | 986 | 1.801 | 369 | 206 | 115 | 136 | 31 | 3.644 |
| **M45-49y** | 1,489 | 2,954 | 749 | 298 | 191 | 151 | 37 | 5,869 |
| **M50-54y** | 2,441 | 4,951 | 1268 | 463 | 267 | 173 | 60 | 9,623 |
| **M55-59y** | 3,393 | 6,712 | 1702 | 752 | 361 | 233 | 62 | 13,215 |
| **M60-64y** | 4,803 | 8,385 | 2328 | 1117 | 519 | 262 | 67 | 17,481 |
| **M65-69y** | 5,990 | 8,999 | 2758 | 1375 | 697 | 291 | 81 | 20,191 |
| **M70-74y** | 6,818 | 8,680 | 3025 | 1595 | 649 | 306 | 67 | 21,140 |
| **M75-79y** | 7,436 | 7,992 | 3164 | 1906 | 655 | 299 | 64 | 21,516 |
| **M>=80y** | 16,178 | 14,623 | 8839 | 5095 | 900 | 749 | 107 | 46,491 |
| **Total** | 50,762 | 67,121 | 24,707 | 13,105 | 4,537 | 2,832 | 689 | 163,753 |
| **F30-34y** | 292 | 175 | 81 | 37 | 9 | 86 | 42 | 722 |
| **F35-39y** | 555 | 389 | 174 | 67 | 43 | 109 | 56 | 1,393 |
| **F40-44y** | 918 | 769 | 344 | 119 | 61 | 145 | 54 | 2,410 |
| **F45-49y** | 1,455 | 1,367 | 580 | 216 | 85 | 197 | 86 | 3,986 |
| **F50-54y** | 2,043 | 2,116 | 852 | 330 | 133 | 185 | 115 | 5,774 |
| **F55-59y** | 2,510 | 2,995 | 1,235 | 492 | 176 | 211 | 128 | 7,747 |
| **F60-64y** | 3,365 | 4,321 | 1,759 | 801 | 293 | 263 | 125 | 10,927 |
| **F65-69y** | 4,341 | 5,265 | 2,189 | 1,043 | 386 | 337 | 131 | 13,692 |
| **F70-74y** | 5,345 | 5,914 | 2,742 | 1,451 | 447 | 424 | 116 | 16,439 |
| **F75-79y** | 6,962 | 6,567 | 3,535 | 1,932 | 489 | 461 | 105 | 20,051 |
| **F>=80y** | 22,028 | 17,788 | 14,257 | 7,732 | 900 | 1577 | 201 | 64,483 |
| **Total** | 50,138 | 47,818 | 27,821 | 14,282 | 3,044 | 4,094 | 1,213 | 148,410 |

**Table 3. Hospitalizations costs from Cardiovascular Diseases to the National Health System in Brazil, 2017 (Brazil Reals - National Hospital Information System – SIH/SUS)**

|  | **I60-I69: Cerebrovascular diseases** | **I20-I25: Ischaemic heart diseases** | **I10-I15: Hypertensive disease** | **I50: Heart failure** | **I71: Aortic aneurysm** | **I26: Pulmonary embolism** | **I05-09: Rheumatic heart disease** | **Total** |
| --- | --- | --- | --- | --- | --- | --- | --- | --- |
| **M30-34y** | 514,891.41 | 835,001.15 | 136,720.61 | 308,858.34 | 25,533.01 | 166,899.00 | 102,470.26 | 2,090,373.79 |
| **M35-39y** | 1,276,483.56 | 1,278,414.02 | 279,251.55 | 445,182.90 | 19,674.37 | 248,040.01 | 142,552.59 | 3,689,599.00 |
| **M40-44y** | 3,000,492.33 | 1,846,974.26 | 362,290.58 | 789,885.28 | 27,260.55 | 349,313.53 | 143,900.07 | 6,520,116.60 |
| **M45-49y** | 6,108,152.51 | 2,582,265.46 | 410,831.55 | 1,041,865.74 | 15,259.31 | 464,858.28 | 169,600.09 | 10,792,832.94 |
| **M50-54y** | 11,061,992.18 | 3,707,598.07 | 451,619.65 | 1,637,214.62 | 23,972.14 | 714,328.62 | 191,048.77 | 17,787,774.05 |
| **M55-59y** | 15,241,196.26 | 4,486,951.47 | 510,983.25 | 2,388,041.13 | 39,030.75 | 1,062,832.00 | 239,184.15 | 23,968,219.00 |
| **M60-64y** | 14,838,981.69 | 4,535,376.27 | 521,653.98 | 2,702,721.78 | 42,335.19 | 1,552,994.98 | 246,986.74 | 24,441,050.63 |
| **M65-69y** | 13,378,758.80 | 4,576,947.99 | 456,235.35 | 2,889,615.54 | 30,882.61 | 1,885,052.12 | 216,160.85 | 23,433,653.25 |
| **M70-74y** | 8,296,482.73 | 3,459,149.28 | 359,549.49 | 2,576,171.19 | 24,075.43 | 1,542,031.70 | 143,141.79 | 16,400,601.63 |
| **M75-79y** | 5,268,661.21 | 2,800,073.67 | 317,291.85 | 2,243,361.05 | 27,985.98 | 1,245,569.85 | 103,678.55 | 12,006,622.17 |
| **M>=80y** | 2,727,618.30 | 2,057,909.02 | 377,847.56 | 2,854,254.78 | 27,463.52 | 841,089.69 | 28,718.19 | 8,914,901.06 |
| **F30-34y** | 188,536.12 | 747,808.24 | 169,429.89 | 200,378.74 | 23,217.93 | 80,369.36 | 136,678.54 | 1,546,418.81 |
| **F35-39y** | 550,232.84 | 1,328,341.34 | 197,570.62 | 341,733.10 | 32,674.83 | 134,428.52 | 198,852.37 | 2,783,833.62 |
| **F40-44y** | 1,247,856.08 | 2,080,061.17 | 316,855.67 | 432,262.56 | 31,386.17 | 169,452.55 | 236,370.25 | 4,514,244.44 |
| **F45-49y** | 2,588,545.44 | 2,960,017.34 | 287,111.29 | 708,874.08 | 39,610.51 | 209,418.23 | 247,260.35 | 7,040,837.24 |
| **F50-54y** | 4,171,861.37 | 3,638,838.24 | 368,175.51 | 1,116,057.97 | 32,600.12 | 312,360.94 | 298,783.63 | 9,938,677.79 |
| **F55-59y** | 6,482,273.60 | 3,875,005.21 | 374,384.89 | 1,522,741.50 | 36,511.15 | 471,712.05 | 277,384.61 | 13,040,013.02 |
| **F60-64y** | 6,703,344.41 | 3,305,171.04 | 417,885.10 | 1,751,258.77 | 44,032.49 | 534,585.73 | 227,965.32 | 12,984,242.85 |
| **F65-69y** | 6,675,216.95 | 3,466,823.24 | 464,751.28 | 1,987,563.32 | 38,476.74 | 656,948.63 | 182,224.30 | 13,472,004.44 |
| **F70-74y** | 4,698,630.18 | 2,541,154.54 | 411,718.90 | 2,057,540.81 | 41,926.87 | 683,330.80 | 149,335.73 | 10,583,637.82 |
| **F75-79y** | 3,348,387.98 | 2,442,392.62 | 324,708.25 | 1,981,882.67 | 42,304.91 | 490,508.12 | 70,379.69 | 8,700,564.24 |
| **F>=80y** | 2,132,609.31 | 1,955,439.27 | 538,004.49 | 3,432,689.63 | 65,821.68 | 343,774.44 | 26,461.74 | 8,494,800.57 |
| **Total** | 120,501,205.26 | 60,507,712.90 | 8,054,871.32 | 35,410,155.48 | 732,036.27 | 14,159,899.14 | 3,779,138.58 | 243,145,018.96 |

**Table 4. Outpatient costs from Cardiovascular Diseases to the National Health System in Brazil, 2017 (Brazil Reals - National Outpatient Care Information on Mortality – SIA/SUS)**

|  | **I60-I69: Cerebrovascular diseases** | **I20-I25: Ischaemic heart diseases** | **I10-I15: Hypertensive disease** | **I50: Heart failure** | **I71: Aortic aneurysm** | **I26: Pulmonary embolism** | **I05-09: Rheumatic heart disease** | **Total** |
| --- | --- | --- | --- | --- | --- | --- | --- | --- |
| **M30-34y** | 50,830.53 | 65,537.77 | 27,492.90 | 9,364.81 | 905.31 | 304.32 | 494.32 | 154,929.97 |
| **M35-39y** | 118,485.09 | 90,836.80 | 43,487.80 | 13,999.70 | 902.64 | 1,197.28 | 1,069.37 | 269,978.68 |
| **M40-44y** | 265,278.69 | 123,748.98 | 61,011.90 | 15,231.34 | 970.27 | 1,060.18 | 1,937.70 | 469,239.06 |
| **M45-49y** | 473,404.36 | 198,164.81 | 82,416.41 | 21,921.50 | 845.32 | 1,625.67 | 3,499.25 | 781,877.31 |
| **M50-54y** | 804,547.91 | 270,395.72 | 127,018.77 | 31,446.36 | 1,003.48 | 2,106.25 | 4,353.54 | 1,240,872.02 |
| **M55-59y** | 1,157,159.32 | 362,532.10 | 170,884.45 | 44,246.62 | 1,203.74 | 3,734.58 | 5,671.19 | 1,745,432.00 |
| **M60-64y** | 1,147,575.67 | 357,614.99 | 190,977.28 | 46,386.35 | 725.02 | 7,182.91 | 5,755.73 | 1,756,217.96 |
| **M65-69y** | 1,034,842.94 | 371,091.46 | 185,395.52 | 47,326.21 | 905.96 | 6,258.88 | 4,788.96 | 1,650,609.94 |
| **M70-74y** | 631,545.52 | 244,421.35 | 145,881.43 | 42,362.51 | 816.88 | 5,098.93 | 4,132.76 | 1,074,259.38 |
| **M75-79y** | 388,504.50 | 198,946.06 | 96,690.01 | 27,496.70 | 787.35 | 5,405.91 | 2,642.15 | 720,472.68 |
| **M>=80y** | 184,653.44 | 120,524.31 | 81,163.92 | 32,632.13 | 799.13 | 3,937.41 | 1,384.10 | 425,094.42 |
| **F30-34y** | 35,751.84 | 65,101.24 | 39,356.51 | 6,403.07 | 221.52 | 257.54 | 1,162.87 | 148,254.60 |
| **F35-39y** | 91,284.09 | 105,087.49 | 61,886.42 | 10,929.52 | 253.93 | 606.84 | 1,621.55 | 271,669.84 |
| **F40-44y** | 223,710.63 | 131,787.92 | 88,709.10 | 13,812.04 | 619.20 | 1,620.14 | 2,790.62 | 463,049.65 |
| **F45-49y** | 431,543.35 | 179,430.36 | 127,056.23 | 18,583.72 | 447.02 | 3,015.75 | 3,718.53 | 763,794.96 |
| **F50-54y** | 699,523.44 | 236,170.76 | 180,814.12 | 27,236.79 | 540.97 | 3,383.47 | 4,843.98 | 1,152,513.53 |
| **F55-59y** | 937,200.31 | 275,512.95 | 217,963.26 | 42,190.38 | 1,124.21 | 4,568.10 | 5,869.40 | 1,484,428.60 |
| **F60-64y** | 918,691.25 | 228,459.80 | 227,558.11 | 45,530.37 | 558.62 | 7,882.66 | 6,087.83 | 1,434,768.64 |
| **F65-69y** | 856,986.27 | 240,831.94 | 217,419.50 | 42,922.71 | 660.64 | 10,224.95 | 4,468.58 | 1,373,514.60 |
| **F70-74y** | 538,721.05 | 162,950.26 | 180,665.31 | 33,722.73 | 382.62 | 9,770.89 | 3,324.69 | 929,537.54 |
| **F75-79y** | 336,835.10 | 137,712.11 | 121,310.79 | 25,730.96 | 312.08 | 6,958.30 | 1,975.33 | 630,834.68 |
| **F>=80y** | 168,121.56 | 107,363.32 | 108,317.38 | 20,914.66 | 374.27 | 5,933.32 | 1,306.99 | 412,331.52 |
| **Total F** | 11,495,196.86 | 4,274,222.52 | 2,783,477.11 | 620,391.21 | 15,360.18 | 92,134.28 | 72,899.44 | 19,353,681.58 |

**Table 5. Costs of hypertension drugs provided by the *Farmacia Popular* Program in Brazil, 2017.**

|  | **Brazil Reals** | **US$** |
| --- | --- | --- |
| **M30-34** | 9,946,729.00 | 3,118,096.87 |
| **M35-39** | 18,555,510.00 | 5,816,774.29 |
| **M40-44** | 28,534,092.00 | 8,944,856.43 |
| **M45-49** | 44,002,948.00 | 13,794,027.59 |
| **M50-54** | 64,285,896.00 | 20,152,318.50 |
| **M55-59** | 78,205,080.00 | 24,515,699.06 |
| **M60-64** | 87,789,920.00 | 27,520,351.10 |
| **M65-69** | 82,101,560.00 | 25,737,166.14 |
| **M70-74** | 61,726,248.00 | 19,349,921.00 |
| **M75-79** | 40,900,708.00 | 12,821,538.56 |
| **M>=80** | 33,951,430.75 | 10,643,081.74 |
|  |  |  |
| **F30-34** | 12,825,487.00 | 4,020,528.84 |
| **F35-39** | 26,811,980.00 | 8,405,009.40 |
| **F40-44** | 46,183,456.00 | 14,477,572.41 |
| **F45-49** | 74,451,208.00 | 23,338,936.68 |
| **F50-54** | 108,198,160.00 | 33,917,918.50 |
| **F55-59** | 124,482,880.00 | 39,022,846.39 |
| **F60-64** | 133,087,464.00 | 41,720,208.15 |
| **F65-69** | 122,692,376.00 | 38,461,559.87 |
| **F70-74** | 93,735,048.00 | 29,384,027.59 |
| **F75-79** | 64,415,640.00 | 20,192,990.60 |
| **F>=80** | 61,933,930.00 | 19,415,025.08 |
|  |  |  |
| **Total** | 1,418,817,750.75 | 444,770,454.78 |

**Table 6A. Average salt consumption and prevalence of salt consumption in Brazil, 2013 (National Health Survey – PNS 2013)**

|  |  | Salt | | SD | |
| --- | --- | --- | --- | --- | --- |
|  |  | Male | Female | Male | Female |
| 18-29y | 9.36 | 9.7 | 9.1 | 3.7 | 3.5 |
| 30-44y | 9.56 | 9.9 | 9.3 | 3.8 | 3.6 |
| 45-59y | 9.28 | 9.6 | 9.0 | 3.7 | 3.5 |
| >=60y | 9.01 | 9.3 | 8.8 | 3.6 | 3.4 |
|  |  |  |  |  |  |
|  | Prevalences |  |  |  |  |
| Male | <5g | 5-8g | 8-12g | >=12g |  |
| 18-29y | 0.01 | 0.22 | 0.62 | 0.15 |  |
| 30-44y | 0.02 | 0.20 | 0.61 | 0.18 |  |
| 45-59y | 0.02 | 0.22 | 0.61 | 0.15 |  |
| >=60y | 0.03 | 0.26 | 0.55 | 0.14 |  |
|  |  |  |  |  |  |
| Female | <5g | 5-8g | 8-12g | >=12g |  |
| 18-29y | 0.02 | 0.30 | 0.58 | 0.10 |  |
| 30-44y | 0.02 | 0.27 | 0.58 | 0.13 |  |
| 45-59y | 0.03 | 0.30 | 0.57 | 0.10 |  |
| >=60y | 0.05 | 0.35 | 0.51 | 0.10 |  |

**Table 6B. Average salt consumption and standard deviation of salt consumption in Brazil at baseline and in the counterfactual scenario, 2013 (National Health Survey – PNS 2013)**

| **Population Characteristics** | | |  |  | | |  |  | |  | |  | |  | |  | |  |
| --- | --- | --- | --- | --- | --- | --- | --- | --- | --- | --- | --- | --- | --- | --- | --- | --- | --- | --- |
|  |  | |  |  | | |  |  | |  | |  | |  | |  | |  |
|  |  | Baseline | | | | | | | | Counterfactual | | | | | | | |  |
|  |  | Mean Salt (g/d) | | | SD Salt (g/d) | | Mean log Salt | | SD log Salt | | Mean Salt (g/d) | | SD Salt (g/d) | | Mean log Salt | | SD log Salt | |
| M30-34 |  | 9.56 | | | 1.1 | | 2.25101 | | 0.11468 | | 5.0 | | 0.549 | | 1.60345 | | 0.10947 | |
| M35-39 |  | 9.56 | | | 1.3 | | 2.24843 | | 0.13536 | | 5.0 | | 0.549 | | 1.60345 | | 0.10947 | |
| M40-44 |  | 9.56 | | | 1.3 | | 2.24843 | | 0.13536 | | 5.0 | | 0.549 | | 1.60345 | | 0.10947 | |
| M45-49 |  | 9.28 | | | 0.8 | | 2.22416 | | 0.08605 | | 5.0 | | 0.549 | | 1.60345 | | 0.10947 | |
| M50-54 |  | 9.28 | | | 0.8 | | 2.22416 | | 0.08605 | | 5.0 | | 0.549 | | 1.60345 | | 0.10947 | |
| M55-59 |  | 9.28 | | | 0.8 | | 2.22416 | | 0.08605 | | 5.0 | | 0.549 | | 1.60345 | | 0.10947 | |
| M60-64 |  | 9.01 | | | 1.0 | | 2.19221 | | 0.11065 | | 5.0 | | 0.549 | | 1.60345 | | 0.10947 | |
| M65-69 |  | 9.01 | | | 1.0 | | 2.19221 | | 0.11065 | | 5.0 | | 0.549 | | 1.60345 | | 0.10947 | |
| M70-74 |  | 9.01 | | | 1.0 | | 2.19221 | | 0.11065 | | 5.0 | | 0.549 | | 1.60345 | | 0.10947 | |
| M75-79 |  | 9.01 | | | 1.0 | | 2.19221 | | 0.11065 | | 5.0 | | 0.549 | | 1.60345 | | 0.10947 | |
| M80-84 |  | 9.01 | | | 1.0 | | 2.19221 | | 0.11065 | | 5.0 | | 0.549 | | 1.60345 | | 0.10947 | |
| M85+ |  | 9.01 | | | 1.0 | | 2.19221 | | 0.11065 | | 5.0 | | 0.549 | | 1.60345 | | 0.10947 | |
|  |  | |  | | |  | |  | |  | | | |  | |  | |  |
|  |  | |  |  | | |  |  | |  | |  | |  | |  | |  |
| F30-34 |  | | 9.56 | 1.1 | | | 2.25101 | 0.11468 | | 5.0 | | 0.549 | | 1.60345 | | 0.10947 | |  |
| F35-39 |  | | 9.56 | 1.3 | | | 2.24843 | 0.13536 | | 5.0 | | 0.549 | | 1.60345 | | 0.10947 | |  |
| F40-44 |  | | 9.56 | 1.3 | | | 2.24843 | 0.13536 | | 5.0 | | 0.549 | | 1.60345 | | 0.10947 | |  |
| F45-49 |  | | 9.28 | 0.8 | | | 2.22416 | 0.08605 | | 5.0 | | 0.549 | | 1.60345 | | 0.10947 | |  |
| F50-54 |  | | 9.28 | 0.8 | | | 2.22416 | 0.08605 | | 5.0 | | 0.549 | | 1.60345 | | 0.10947 | |  |
| F55-59 |  | | 9.28 | 0.8 | | | 2.22416 | 0.08605 | | 5.0 | | 0.549 | | 1.60345 | | 0.10947 | |  |
| F60-64 |  | | 9.01 | 1.0 | | | 2.19221 | 0.11065 | | 5.0 | | 0.549 | | 1.60345 | | 0.10947 | |  |
| F65-69 |  | | 9.01 | 1.0 | | | 2.19221 | 0.11065 | | 5.0 | | 0.549 | | 1.60345 | | 0.10947 | |  |
| F70-74 |  | | 9.01 | 1.0 | | | 2.19221 | 0.11065 | | 5.0 | | 0.549 | | 1.60345 | | 0.10947 | |  |
| F75-79 |  | | 9.01 | 1.0 | | | 2.19221 | 0.11065 | | 5.0 | | 0.549 | | 1.60345 | | 0.10947 | |  |
| F80-84 |  | | 9.01 | 1.0 | | | 2.19221 | 0.11065 | | 5.0 | | 0.549 | | 1.60345 | | 0.10947 | |  |
| F85+ |  | | 9.01 | 1.0 | | | 2.19221 | 0.11065 | | 5.0 | | 0.549 | | 1.60345 | | 0.10947 | |  |

**Table 6C. Log-normal distribution of salt consumption in Brazil at baseline (Brazil, 2013)**

|  | Baseline salt distribution (g/day) | | | |  |  |  |  |  |  |  |  |  |  |  |  |  |  |  |  |  |  |  |  |  |
| --- | --- | --- | --- | --- | --- | --- | --- | --- | --- | --- | --- | --- | --- | --- | --- | --- | --- | --- | --- | --- | --- | --- | --- | --- | --- |
|  | <1 | 1-2 | 2-3 | 3-4 | 4-5 | 5-6 | 6-7 | 7-8 | 8-9 | 9-10 | 10-11 | 11-12 | 12-13 | 13-14 | 14-15 | 15-16 | 16-17 | 17-18 | 18-19 | 19-20 | 20-21 | 21-22 | 22-23 | 23-24 | 24+ |
| M30-34 | 0 | 0 | 0 | 0 | 0 | 274 | 34136 | 559150 | 2223577 | 3120997 | 1995361 | 700341 | 155216 | 24145 | 2857 | 273 | 22 | 2 | 0 | 0 | 0 | 0 | 0 | 0 | 0 |
| M35-39 | 0 | 0 | 0 | 0 | 9 | 2913 | 97247 | 734613 | 1943717 | 2386288 | 1653080 | 744091 | 241364 | 60928 | 12681 | 2274 | 363 | 53 | 7 | 1 | 0 | 0 | 0 | 0 | 0 |
| M40-44 | 0 | 0 | 0 | 0 | 8 | 2544 | 84937 | 641626 | 1697682 | 2084233 | 1443833 | 649904 | 210812 | 53216 | 11076 | 1986 | 317 | 46 | 6 | 1 | 0 | 0 | 0 | 0 | 0 |
| M45-49 | 0 | 0 | 0 | 0 | 0 | 2 | 3827 | 286291 | 2073018 | 2768563 | 998164 | 128569 | 7421 | 230 | 4 | 0 | 0 | 0 | 0 | 0 | 0 | 0 | 0 | 0 | 0 |
| M50-54 | 0 | 0 | 0 | 0 | 0 | 1 | 3457 | 258581 | 1872373 | 2500597 | 901553 | 116125 | 6702 | 208 | 4 | 0 | 0 | 0 | 0 | 0 | 0 | 0 | 0 | 0 | 0 |
| M55-59 | 0 | 0 | 0 | 0 | 0 | 1 | 2858 | 213765 | 1547861 | 2067204 | 745299 | 95999 | 5541 | 172 | 3 | 0 | 0 | 0 | 0 | 0 | 0 | 0 | 0 | 0 | 0 |
| M60-64 | 0 | 0 | 0 | 0 | 0 | 540 | 47001 | 515536 | 1330449 | 1179395 | 466889 | 100296 | 13537 | 1282 | 93 | 5 | 0 | 0 | 0 | 0 | 0 | 0 | 0 | 0 | 0 |
| M65-69 | 0 | 0 | 0 | 0 | 0 | 395 | 34360 | 376888 | 972639 | 862209 | 341324 | 73323 | 9897 | 937 | 68 | 4 | 0 | 0 | 0 | 0 | 0 | 0 | 0 | 0 | 0 |
| M70-74 | 0 | 0 | 0 | 0 | 0 | 265 | 23064 | 252977 | 652860 | 578736 | 229105 | 49216 | 6643 | 629 | 45 | 3 | 0 | 0 | 0 | 0 | 0 | 0 | 0 | 0 | 0 |
| M75-79 | 0 | 0 | 0 | 0 | 0 | 181 | 15718 | 172402 | 444919 | 394405 | 156134 | 33540 | 4527 | 429 | 31 | 2 | 0 | 0 | 0 | 0 | 0 | 0 | 0 | 0 | 0 |
| M80-84 | 0 | 0 | 0 | 0 | 0 | 92 | 8016 | 87928 | 226917 | 201153 | 79631 | 17106 | 2309 | 219 | 16 | 1 | 0 | 0 | 0 | 0 | 0 | 0 | 0 | 0 | 0 |
| M85+ | 0 | 0 | 0 | 0 | 0 | 92 | 8016 | 87928 | 226917 | 201153 | 79631 | 17106 | 2309 | 219 | 16 | 1 | 0 | 0 | 0 | 0 | 0 | 0 | 0 | 0 | 0 |
|  |  |  |  |  |  |  |  |  |  |  |  |  |  |  |  |  |  |  |  |  |  |  |  |  |  |
| F30-34 | 0 | 0 | 0 | 0 | 0 | 274 | 34154 | 559449 | 2224767 | 3122667 | 1996428 | 700716 | 155300 | 24157 | 2858 | 274 | 22 | 2 | 0 | 0 | 0 | 0 | 0 | 0 | 0 |
| F35-39 | 0 | 0 | 0 | 0 | 9 | 2949 | 98444 | 743655 | 1967643 | 2415661 | 1673427 | 753250 | 244335 | 61678 | 12837 | 2302 | 368 | 54 | 7 | 1 | 0 | 0 | 0 | 0 | 0 |
| F40-44 | 0 | 0 | 0 | 0 | 8 | 2610 | 87156 | 658386 | 1742028 | 2138675 | 1481548 | 666880 | 216319 | 54606 | 11365 | 2038 | 326 | 48 | 6 | 1 | 0 | 0 | 0 | 0 | 0 |
| F45-49 | 0 | 0 | 0 | 0 | 0 | 2 | 3992 | 298637 | 2162416 | 2887956 | 1041209 | 134114 | 7741 | 240 | 5 | 0 | 0 | 0 | 0 | 0 | 0 | 0 | 0 | 0 | 0 |
| F50-54 | 0 | 0 | 0 | 0 | 0 | 2 | 3682 | 275399 | 1994153 | 2663236 | 960190 | 123678 | 7138 | 221 | 4 | 0 | 0 | 0 | 0 | 0 | 0 | 0 | 0 | 0 | 0 |
| F55-59 | 0 | 0 | 0 | 0 | 0 | 1 | 3128 | 233968 | 1694151 | 2262577 | 815738 | 105072 | 6064 | 188 | 4 | 0 | 0 | 0 | 0 | 0 | 0 | 0 | 0 | 0 | 0 |
| F60-64 | 0 | 0 | 0 | 0 | 0 | 612 | 53263 | 584221 | 1507705 | 1336526 | 529093 | 113659 | 15341 | 1453 | 105 | 6 | 0 | 0 | 0 | 0 | 0 | 0 | 0 | 0 | 0 |
| F65-69 | 0 | 0 | 0 | 0 | 0 | 469 | 40798 | 447499 | 1154865 | 1023746 | 405272 | 87060 | 11751 | 1113 | 80 | 5 | 0 | 0 | 0 | 0 | 0 | 0 | 0 | 0 | 0 |
| F70-74 | 0 | 0 | 0 | 0 | 0 | 337 | 29358 | 322017 | 831032 | 736680 | 291631 | 62648 | 8456 | 801 | 58 | 3 | 0 | 0 | 0 | 0 | 0 | 0 | 0 | 0 | 0 |
| F75-79 | 0 | 0 | 0 | 0 | 0 | 250 | 21749 | 238553 | 615636 | 545739 | 216043 | 46410 | 6264 | 593 | 43 | 3 | 0 | 0 | 0 | 0 | 0 | 0 | 0 | 0 | 0 |
| F80-84 | 0 | 0 | 0 | 0 | 0 | 152 | 13259 | 145435 | 375326 | 332713 | 131712 | 28294 | 3819 | 362 | 26 | 2 | 0 | 0 | 0 | 0 | 0 | 0 | 0 | 0 | 0 |
| F85+ | 0 | 0 | 0 | 0 | 0 | 152 | 13259 | 145435 | 375326 | 332713 | 131712 | 28294 | 3819 | 362 | 26 | 2 | 0 | 0 | 0 | 0 | 0 | 0 | 0 | 0 | 0 |

**Table 6D. Log-normal distribution of salt consumption in Brazil in the counterfactual scenario (Brazil, 2013)**

|  | Counterfactual salt distribution (g/day) | | | |  |  |  |  |  |  |  |  |  |  |  |  |  |  |  |  |  |  |  |  |  |
| --- | --- | --- | --- | --- | --- | --- | --- | --- | --- | --- | --- | --- | --- | --- | --- | --- | --- | --- | --- | --- | --- | --- | --- | --- | --- |
|  | <1 | 1-2 | 2-3 | 3-4 | 4-5 | 5-6 | 6-7 | 7-8 | 8-9 | 9-10 | 10-11 | 11-12 | 12-13 | 13-14 | 14-15 | 15-16 | 16-17 | 17-18 | 18-19 | 19-20 | 20-21 | 21-22 | 22-23 | 23-24 | 24+ |
| M30-34 | 0 | 0 | 18 | 208471 | 4392107 | 3839322 | 368683 | 7689 | 60 | 0 | 0 | 0 | 0 | 0 | 0 | 0 | 0 | 0 | 0 | 0 | 0 | 0 | 0 | 0 | 0 |
| M35-39 | 0 | 0 | 16 | 186321 | 3925454 | 3431401 | 329511 | 6872 | 54 | 0 | 0 | 0 | 0 | 0 | 0 | 0 | 0 | 0 | 0 | 0 | 0 | 0 | 0 | 0 | 0 |
| M40-44 | 0 | 0 | 14 | 162737 | 3428572 | 2997056 | 287802 | 6002 | 47 | 0 | 0 | 0 | 0 | 0 | 0 | 0 | 0 | 0 | 0 | 0 | 0 | 0 | 0 | 0 | 0 |
| M45-49 | 0 | 0 | 13 | 148168 | 3121624 | 2728740 | 262036 | 5465 | 43 | 0 | 0 | 0 | 0 | 0 | 0 | 0 | 0 | 0 | 0 | 0 | 0 | 0 | 0 | 0 | 0 |
| M50-54 | 0 | 0 | 11 | 133827 | 2819486 | 2464629 | 236674 | 4936 | 39 | 0 | 0 | 0 | 0 | 0 | 0 | 0 | 0 | 0 | 0 | 0 | 0 | 0 | 0 | 0 | 0 |
| M55-59 | 0 | 0 | 9 | 110632 | 2330824 | 2037469 | 195655 | 4080 | 32 | 0 | 0 | 0 | 0 | 0 | 0 | 0 | 0 | 0 | 0 | 0 | 0 | 0 | 0 | 0 | 0 |
| M60-64 | 0 | 0 | 7 | 86426 | 1820851 | 1591681 | 152846 | 3188 | 25 | 0 | 0 | 0 | 0 | 0 | 0 | 0 | 0 | 0 | 0 | 0 | 0 | 0 | 0 | 0 | 0 |
| M65-69 | 0 | 0 | 5 | 63183 | 1331152 | 1163615 | 111740 | 2330 | 18 | 0 | 0 | 0 | 0 | 0 | 0 | 0 | 0 | 0 | 0 | 0 | 0 | 0 | 0 | 0 | 0 |
| M70-74 | 0 | 0 | 4 | 42410 | 893503 | 781048 | 75003 | 1564 | 12 | 0 | 0 | 0 | 0 | 0 | 0 | 0 | 0 | 0 | 0 | 0 | 0 | 0 | 0 | 0 | 0 |
| M75-79 | 0 | 0 | 2 | 28902 | 608915 | 532278 | 51114 | 1066 | 8 | 0 | 0 | 0 | 0 | 0 | 0 | 0 | 0 | 0 | 0 | 0 | 0 | 0 | 0 | 0 | 0 |
| M80-84 | 0 | 0 | 1 | 14741 | 310558 | 271471 | 26069 | 544 | 4 | 0 | 0 | 0 | 0 | 0 | 0 | 0 | 0 | 0 | 0 | 0 | 0 | 0 | 0 | 0 | 0 |
| M85+ | 0 | 0 | 1 | 14741 | 310558 | 271471 | 26069 | 544 | 4 | 0 | 0 | 0 | 0 | 0 | 0 | 0 | 0 | 0 | 0 | 0 | 0 | 0 | 0 | 0 | 0 |
|  |  |  |  |  |  |  |  |  |  |  |  |  |  |  |  |  |  |  |  |  |  |  |  |  |  |
| F30-34 | 0 | 0 | 18 | 208582 | 4394457 | 3841376 | 368881 | 7693 | 60 | 0 | 0 | 0 | 0 | 0 | 0 | 0 | 0 | 0 | 0 | 0 | 0 | 0 | 0 | 0 | 0 |
| F35-39 | 0 | 0 | 16 | 188615 | 3973773 | 3473638 | 333567 | 6956 | 55 | 0 | 0 | 0 | 0 | 0 | 0 | 0 | 0 | 0 | 0 | 0 | 0 | 0 | 0 | 0 | 0 |
| F40-44 | 0 | 0 | 14 | 166988 | 3518130 | 3075342 | 295320 | 6159 | 48 | 0 | 0 | 0 | 0 | 0 | 0 | 0 | 0 | 0 | 0 | 0 | 0 | 0 | 0 | 0 | 0 |
| F45-49 | 0 | 0 | 13 | 154557 | 3256243 | 2846416 | 273336 | 5700 | 45 | 0 | 0 | 0 | 0 | 0 | 0 | 0 | 0 | 0 | 0 | 0 | 0 | 0 | 0 | 0 | 0 |
| F50-54 | 0 | 0 | 12 | 142531 | 3002866 | 2624929 | 252067 | 5257 | 41 | 0 | 0 | 0 | 0 | 0 | 0 | 0 | 0 | 0 | 0 | 0 | 0 | 0 | 0 | 0 | 0 |
| F55-59 | 0 | 0 | 10 | 121088 | 2551112 | 2230032 | 214146 | 4466 | 35 | 0 | 0 | 0 | 0 | 0 | 0 | 0 | 0 | 0 | 0 | 0 | 0 | 0 | 0 | 0 | 0 |
| F60-64 | 0 | 0 | 8 | 97941 | 2063443 | 1803741 | 173210 | 3612 | 28 | 0 | 0 | 0 | 0 | 0 | 0 | 0 | 0 | 0 | 0 | 0 | 0 | 0 | 0 | 0 | 0 |
| F65-69 | 0 | 0 | 6 | 75020 | 1580546 | 1381621 | 132675 | 2767 | 22 | 0 | 0 | 0 | 0 | 0 | 0 | 0 | 0 | 0 | 0 | 0 | 0 | 0 | 0 | 0 | 0 |
| F70-74 | 0 | 0 | 5 | 53984 | 1137350 | 994204 | 95472 | 1991 | 16 | 0 | 0 | 0 | 0 | 0 | 0 | 0 | 0 | 0 | 0 | 0 | 0 | 0 | 0 | 0 | 0 |
| F75-79 | 0 | 0 | 3 | 39992 | 842559 | 736515 | 70726 | 1475 | 12 | 0 | 0 | 0 | 0 | 0 | 0 | 0 | 0 | 0 | 0 | 0 | 0 | 0 | 0 | 0 | 0 |
| F80-84 | 0 | 0 | 2 | 24381 | 513670 | 449020 | 43119 | 899 | 7 | 0 | 0 | 0 | 0 | 0 | 0 | 0 | 0 | 0 | 0 | 0 | 0 | 0 | 0 | 0 | 0 |
| F85+ | 0 | 0 | 2 | 24381 | 513670 | 449020 | 43119 | 899 | 7 | 0 | 0 | 0 | 0 | 0 | 0 | 0 | 0 | 0 | 0 | 0 | 0 | 0 | 0 | 0 | 0 |

**Table 7. Summary of the relative risks of salt consumption to blood pressure and of blood pressure to cardiovascular disease outcomes used for the health and economic modeling.**

| **Model inputs** | **Value** | **Source** |
| --- | --- | --- |
| Effect of salt consumption on systolic blood pressure | -5.80 (-2.50, 9.20) | (16) |
| Relative risk of systolic blood pressure | Unit of change: 20 mmHg SBP decrease | (19) |
| Coronary heart disease | <49 y: 0.49 (0.45–0.53)  50–59 y: 0.50 (0.49–0.52)  60–69 y: 0.54 (0.53–0.55)  70–79 y: 0.60 (0.58–0.61)  Over 79 y: 0.67 (0.64–0.70) | (19) |
| Stroke | <49 y: 0.36 (0.32–0.40)  50–59 y: 0.38 (0.35–0.40)  60–69 y: 0.43 (0.41–0.45)  70–79 y: 0.50 (0.48–0.52)  Over 79 y: 0.67 (0.63–0.71) | (19) |
| Hypertensive disease | 0.22 (0.20–0.25) | (19) |
| Heart failure | 0.53 (0.48–0.59) | (19) |
| Pulmonary embolism | 0.72 (0.60–0.87) | (19) |
| Rheumatic heart disease | 0.74 (0.61–0.89) | (19) |
| Aortic aneurysm | 0.55 (0.49–0.62) | (19) |

**Table 8. Relative risks for coronary heart disease (CHD) and stroke by age group and range of salt consumption - Brazil, 2017**

| **CHD** |  |  |  |  |  |  |  |  |  |  |  |  |  |  |  |
| --- | --- | --- | --- | --- | --- | --- | --- | --- | --- | --- | --- | --- | --- | --- | --- |
|  | **<5g** | **5-6g** | **6-7g** | **7-8g** | **8-9g** | **9-10g** | **10-11g** | **11-12g** | **12-13g** | **13-14g** | **14-15g** | **15-16g** | **16-17g** | **17-18g** | **>=18g** |
| **<50y** | 1.00 | 1.02 | 1.04 | 1.06 | 1.08 | 1.11 | 1.13 | 1.15 | 1.17 | 1.20 | 1.22 | 1.25 | 1.27 | 1.30 | 1.32 |
| **50-59y** | 1.00 | 1.02 | 1.04 | 1.06 | 1.09 | 1.11 | 1.13 | 1.16 | 1.18 | 1.21 | 1.23 | 1.26 | 1.29 | 1.31 | 1.34 |
| **60-69y** | 1.00 | 1.02 | 1.04 | 1.05 | 1.07 | 1.09 | 1.11 | 1.13 | 1.15 | 1.17 | 1.19 | 1.21 | 1.23 | 1.25 | 1.27 |
| **70-79y** | 1.00 | 1.01 | 1.03 | 1.04 | 1.06 | 1.08 | 1.09 | 1.11 | 1.12 | 1.14 | 1.16 | 1.17 | 1.19 | 1.21 | 1.22 |
| **>=80y** | 1.00 | 1.01 | 1.02 | 1.03 | 1.05 | 1.06 | 1.07 | 1.08 | 1.09 | 1.11 | 1.12 | 1.13 | 1.14 | 1.16 | 1.17 |
|  |  |  |  |  |  |  |  |  |  |  |  |  |  |  |  |
| **Stroke** |  |  |  |  |  |  |  |  |  |  |  |  |  |  |  |
|  | **<5g** | **5-6g** | **6-7g** | **7-8g** | **8-9g** | **9-10g** | **10-11g** | **11-12g** | **12-13g** | **13-14g** | **14-15g** | **15-16g** | **16-17g** | **17-18g** | **>=18g** |
| **<50y** | 1.00 | 1.02 | 1.04 | 1.06 | 1.08 | 1.11 | 1.13 | 1.15 | 1.17 | 1.20 | 1.22 | 1.25 | 1.27 | 1.30 | 1.32 |
| **50-59y** | 1.00 | 1.02 | 1.04 | 1.06 | 1.09 | 1.11 | 1.13 | 1.16 | 1.18 | 1.21 | 1.23 | 1.26 | 1.29 | 1.31 | 1.34 |
| **60-69y** | 1.00 | 1.02 | 1.04 | 1.05 | 1.07 | 1.09 | 1.11 | 1.13 | 1.15 | 1.17 | 1.19 | 1.21 | 1.23 | 1.25 | 1.27 |
| **70-79y** | 1.00 | 1.01 | 1.03 | 1.04 | 1.06 | 1.08 | 1.09 | 1.11 | 1.12 | 1.14 | 1.16 | 1.17 | 1.19 | 1.21 | 1.22 |
| **>=80y** | 1.00 | 1.01 | 1.02 | 1.03 | 1.05 | 1.06 | 1.07 | 1.08 | 1.09 | 1.11 | 1.12 | 1.13 | 1.14 | 1.16 | 1.17 |

**Table 9. Relative risks for hypertensive disease, heart failure, pulmonary embolism, rheumatic heart disease and aortic embolism by range of salt consumption - - Brazil, 2017**

|  | **<5g** | **5-6g** | **6-7g** | **7-8g** | **8-9g** | **9-10g** | **10-11g** | **11-12g** | **12-13g** | **13-14g** | **14-15g** | **15-16g** | **16-17g** | **17-18g** | **>=18g** |
| --- | --- | --- | --- | --- | --- | --- | --- | --- | --- | --- | --- | --- | --- | --- | --- |
| **Hypertensive disease** | 1.00 | 1.09 | 1.18 | 1.28 | 1.39 | 1.51 | 1.63 | 1.77 | 1.93 | 2.09 | 2.27 | 2.46 | 2.67 | 2.90 | 3.15 |
| **Heart failure** | 1.00 | 1.04 | 1.08 | 1.12 | 1.17 | 1.21 | 1.26 | 1.31 | 1.37 | 1.42 | 1.48 | 1.53 | 1.60 | 1.66 | 1.72 |
| **Pulmonary embolism** | 1.00 | 1.02 | 1.04 | 1.06 | 1.08 | 1.10 | 1.12 | 1.14 | 1.16 | 1.18 | 1.21 | 1.23 | 1.25 | 1.28 | 1.30 |
| **Rheumatic heart disease** | 1.00 | 1.02 | 1.04 | 1.06 | 1.07 | 1.09 | 1.11 | 1.13 | 1.15 | 1.18 | 1.20 | 1.22 | 1.24 | 1.26 | 1.29 |
| **Aortic aneurysm** | 1.00 | 1.02 | 1.04 | 1.06 | 1.08 | 1.10 | 1.13 | 1.15 | 1.17 | 1.19 | 1.22 | 1.24 | 1.27 | 1.29 | 1.32 |

**Table 10A – Estimated relative risks for Coronary Heart Disease (CHD) and Stroke, by sex, age group and salt consumption interval. Brazil 2013.**

|  | **Relative risks - Salt-CHD** | | | | **Relative risks - Salt-Stroke** | | | |
| --- | --- | --- | --- | --- | --- | --- | --- | --- |
|  | **<5g/d** | **5-8g/d** | **8-12g/d** | **>=12g/d** | **<5g/d** | **5-8g/d** | **8-12g/d** | **>=12g/d** |
| **Men** |  |  |  |  |  |  |  |  |
| **30-34y** | 1.0000 | 1.0351 | 1.1479 | 1.3176 | 1.0000 | 1.0759 | 1.2184 | 1.4845 |
| **35-39y** | 1.0000 | 1.0351 | 1.1479 | 1.3176 | 1.0000 | 1.0759 | 1.2184 | 1.4845 |
| **40-44y** | 1.0000 | 1.0351 | 1.1479 | 1.3176 | 1.0000 | 1.0759 | 1.2184 | 1.4845 |
| **45-49y** | 1.0000 | 1.0351 | 1.1479 | 1.3176 | 1.0000 | 1.0759 | 1.2184 | 1.4845 |
| **50-54y** | 1.0000 | 1.0341 | 1.1434 | 1.3073 | 1.0000 | 1.0759 | 1.2057 | 1.4537 |
| **55-59y** | 1.0000 | 1.0341 | 1.1434 | 1.3073 | 1.0000 | 1.0759 | 1.2057 | 1.4537 |
| **60-64y** | 1.0000 | 1.0302 | 1.1265 | 1.2690 | 1.0000 | 1.0759 | 1.1772 | 1.3859 |
| **65-69y** | 1.0000 | 1.0302 | 1.1265 | 1.2690 | 1.0000 | 1.0759 | 1.1772 | 1.3859 |
| **70-74y** | 1.0000 | 1.0250 | 1.1038 | 1.2184 | 1.0000 | 1.0759 | 1.1434 | 1.3073 |
| **75-79y** | 1.0000 | 1.0250 | 1.1038 | 1.2184 | 1.0000 | 1.0759 | 1.1434 | 1.3073 |
| **>=80y** | 1.0000 | 1.0195 | 1.0805 | 1.1675 | 1.0000 | 1.0759 | 1.0805 | 1.1675 |
|  |  |  |  |  |  |  |  |  |
| **Women** |  |  |  |  |  |  |  |  |
| **30-34y** | 1.0000 | 1.0351 | 1.1479 | 1.3176 | 1.0000 | 1.0506 | 1.2184 | 1.4845 |
| **35-39y** | 1.0000 | 1.0351 | 1.1479 | 1.3176 | 1.0000 | 1.0506 | 1.2184 | 1.4845 |
| **40-44y** | 1.0000 | 1.0351 | 1.1479 | 1.3176 | 1.0000 | 1.0506 | 1.2184 | 1.4845 |
| **45-49y** | 1.0000 | 1.0351 | 1.1479 | 1.3176 | 1.0000 | 1.0506 | 1.2184 | 1.4845 |
| **50-54y** | 1.0000 | 1.0341 | 1.1434 | 1.3073 | 1.0000 | 1.0479 | 1.2057 | 1.4537 |
| **55-59y** | 1.0000 | 1.0341 | 1.1434 | 1.3073 | 1.0000 | 1.0479 | 1.2057 | 1.4537 |
| **60-64y** | 1.0000 | 1.0302 | 1.1265 | 1.2690 | 1.0000 | 1.0416 | 1.1772 | 1.3859 |
| **65-69y** | 1.0000 | 1.0302 | 1.1265 | 1.2690 | 1.0000 | 1.0416 | 1.1772 | 1.3859 |
| **70-74y** | 1.0000 | 1.0250 | 1.1038 | 1.2184 | 1.0000 | 1.0341 | 1.1434 | 1.3073 |
| **75-79y** | 1.0000 | 1.0250 | 1.1038 | 1.2184 | 1.0000 | 1.0341 | 1.1434 | 1.3073 |
| **>=80y** | 1.0000 | 1.0195 | 1.0805 | 1.1675 | 1.0000 | 1.0195 | 1.0805 | 1.1675 |

**Table 10B – Estimated relative risks for hypertensive disease and heart failure, by sex, age group and salt consumption interval. Brazil 2013.**

|  | **Relative risks - Salt-Hypertensive disease** | | | | **Relative risks – Salt – Heart**  **failure** | | | |
| --- | --- | --- | --- | --- | --- | --- | --- | --- |
|  | **<5g/d** | **5-8g/d** | **8-12g/d** | **>=12g/d** | **<5g/d** | **5-8g/d** | **8-12g/d** | **>=12g/d** |
| **Men** |  |  |  |  |  |  |  |  |
| **30-34y** | 1.0000 | 1.0351 | 1.3401 | 1.7958 | 1.0000 | 1.0312 | 1.1306 | 1.2783 |
| **35-39y** | 1.0000 | 1.0351 | 1.3401 | 1.7958 | 1.0000 | 1.0312 | 1.1306 | 1.2783 |
| **40-44y** | 1.0000 | 1.0351 | 1.3401 | 1.7958 | 1.0000 | 1.0312 | 1.1306 | 1.2783 |
| **45-49y** | 1.0000 | 1.0351 | 1.3401 | 1.7958 | 1.0000 | 1.0312 | 1.1306 | 1.2783 |
| **50-54y** | 1.0000 | 1.0351 | 1.3401 | 1.7958 | 1.0000 | 1.0312 | 1.1306 | 1.2783 |
| **55-59y** | 1.0000 | 1.0351 | 1.3401 | 1.7958 | 1.0000 | 1.0312 | 1.1306 | 1.2783 |
| **60-64y** | 1.0000 | 1.0351 | 1.3401 | 1.7958 | 1.0000 | 1.0312 | 1.1306 | 1.2783 |
| **65-69y** | 1.0000 | 1.0351 | 1.3401 | 1.7958 | 1.0000 | 1.0312 | 1.1306 | 1.2783 |
| **70-74y** | 1.0000 | 1.0351 | 1.3401 | 1.7958 | 1.0000 | 1.0312 | 1.1306 | 1.2783 |
| **75-79y** | 1.0000 | 1.0351 | 1.3401 | 1.7958 | 1.0000 | 1.0312 | 1.1306 | 1.2783 |
| **>=80y** | 1.0000 | 1.0351 | 1.3401 | 1.7958 | 1.0000 | 1.0312 | 1.1306 | 1.2783 |
|  |  |  |  |  |  |  |  |  |
| **Women** |  |  |  |  |  |  |  |  |
| **30-34y** | 1.0000 | 1.0351 | 1.3401 | 1.7958 | 1.0000 | 1.0312 | 1.1306 | 1.2783 |
| **35-39y** | 1.0000 | 1.0351 | 1.3401 | 1.7958 | 1.0000 | 1.0312 | 1.1306 | 1.2783 |
| **40-44y** | 1.0000 | 1.0351 | 1.3401 | 1.7958 | 1.0000 | 1.0312 | 1.1306 | 1.2783 |
| **45-49y** | 1.0000 | 1.0351 | 1.3401 | 1.7958 | 1.0000 | 1.0312 | 1.1306 | 1.2783 |
| **50-54y** | 1.0000 | 1.0351 | 1.3401 | 1.7958 | 1.0000 | 1.0312 | 1.1306 | 1.2783 |
| **55-59y** | 1.0000 | 1.0351 | 1.3401 | 1.7958 | 1.0000 | 1.0312 | 1.1306 | 1.2783 |
| **60-64y** | 1.0000 | 1.0351 | 1.3401 | 1.7958 | 1.0000 | 1.0312 | 1.1306 | 1.2783 |
| **65-69y** | 1.0000 | 1.0351 | 1.3401 | 1.7958 | 1.0000 | 1.0312 | 1.1306 | 1.2783 |
| **70-74y** | 1.0000 | 1.0351 | 1.3401 | 1.7958 | 1.0000 | 1.0312 | 1.1306 | 1.2783 |
| **75-79y** | 1.0000 | 1.0351 | 1.3401 | 1.7958 | 1.0000 | 1.0312 | 1.1306 | 1.2783 |
| **>=80y** | 1.0000 | 1.0351 | 1.3401 | 1.7958 | 1.0000 | 1.0312 | 1.1306 | 1.2783 |

**Table 10C – Estimated relative risks for pulmonary embolism and aortic aneurysm, by sex, age group and salt consumption interval. Brazil 2013.**

|  | **Relative risks - Salt- Pulmonary embolism** | | | | **Relative risks – Aortic**  **aneurysm** | | | |
| --- | --- | --- | --- | --- | --- | --- | --- | --- |
|  | **<5g/d** | **5-8g/d** | **8-12g/d** | **>=12g/d** | **<5g/d** | **5-8g/d** | **8-12g/d** | **>=12g/d** |
| **Men** |  |  |  |  |  |  |  |  |
| **30-34y** | 1.0000 | 1.0160 | 1.0656 | 1.1354 | 1.0000 | 1.0351 | 1.1225 | 1.2600 |
| **35-39y** | 1.0000 | 1.0160 | 1.0656 | 1.1354 | 1.0000 | 1.0351 | 1.1225 | 1.2600 |
| **40-44y** | 1.0000 | 1.0160 | 1.0656 | 1.1354 | 1.0000 | 1.0351 | 1.1225 | 1.2600 |
| **45-49y** | 1.0000 | 1.0160 | 1.0656 | 1.1354 | 1.0000 | 1.0351 | 1.1225 | 1.2600 |
| **50-54y** | 1.0000 | 1.0160 | 1.0656 | 1.1354 | 1.0000 | 1.0351 | 1.1225 | 1.2600 |
| **55-59y** | 1.0000 | 1.0160 | 1.0656 | 1.1354 | 1.0000 | 1.0351 | 1.1225 | 1.2600 |
| **60-64y** | 1.0000 | 1.0160 | 1.0656 | 1.1354 | 1.0000 | 1.0351 | 1.1225 | 1.2600 |
| **65-69y** | 1.0000 | 1.0160 | 1.0656 | 1.1354 | 1.0000 | 1.0351 | 1.1225 | 1.2600 |
| **70-74y** | 1.0000 | 1.0160 | 1.0656 | 1.1354 | 1.0000 | 1.0351 | 1.1225 | 1.2600 |
| **75-79y** | 1.0000 | 1.0160 | 1.0656 | 1.1354 | 1.0000 | 1.0351 | 1.1225 | 1.2600 |
| **>=80y** | 1.0000 | 1.0160 | 1.0656 | 1.1354 | 1.0000 | 1.0351 | 1.1225 | 1.2600 |
|  |  |  |  |  |  |  |  |  |
| **Women** |  |  |  |  |  |  |  |  |
| **30-34y** | 1.0000 | 1.0160 | 1.0656 | 1.1354 | 1.0000 | 1.0351 | 1.1225 | 1.2600 |
| **35-39y** | 1.0000 | 1.0160 | 1.0656 | 1.1354 | 1.0000 | 1.0351 | 1.1225 | 1.2600 |
| **40-44y** | 1.0000 | 1.0160 | 1.0656 | 1.1354 | 1.0000 | 1.0351 | 1.1225 | 1.2600 |
| **45-49y** | 1.0000 | 1.0160 | 1.0656 | 1.1354 | 1.0000 | 1.0351 | 1.1225 | 1.2600 |
| **50-54y** | 1.0000 | 1.0160 | 1.0656 | 1.1354 | 1.0000 | 1.0351 | 1.1225 | 1.2600 |
| **55-59y** | 1.0000 | 1.0160 | 1.0656 | 1.1354 | 1.0000 | 1.0351 | 1.1225 | 1.2600 |
| **60-64y** | 1.0000 | 1.0160 | 1.0656 | 1.1354 | 1.0000 | 1.0351 | 1.1225 | 1.2600 |
| **65-69y** | 1.0000 | 1.0160 | 1.0656 | 1.1354 | 1.0000 | 1.0351 | 1.1225 | 1.2600 |
| **70-74y** | 1.0000 | 1.0160 | 1.0656 | 1.1354 | 1.0000 | 1.0351 | 1.1225 | 1.2600 |
| **75-79y** | 1.0000 | 1.0160 | 1.0656 | 1.1354 | 1.0000 | 1.0351 | 1.1225 | 1.2600 |
| **>=80y** | 1.0000 | 1.0160 | 1.0656 | 1.1354 | 1.0000 | 1.0351 | 1.1225 | 1.2600 |

**Table 10D – Estimated relative risks for pulmonary embolism and aortic aneurysm, by sex, age group and salt consumption interval. Brazil 2013.**

|  | **Relative risks - Salt- Rheumatic heart disease** | | | |
| --- | --- | --- | --- | --- |
|  | **<5g/d** | **5-8g/d** | **8-12g/d** | **>=12g/d** |
| **Men** |  |  |  |  |
| **30-34y** | 1.0000 | 1.0147 | 1.0599 | 1.1235 |
| **35-39y** | 1.0000 | 1.0147 | 1.0599 | 1.1235 |
| **40-44y** | 1.0000 | 1.0147 | 1.0599 | 1.1235 |
| **45-49y** | 1.0000 | 1.0147 | 1.0599 | 1.1235 |
| **50-54y** | 1.0000 | 1.0147 | 1.0599 | 1.1235 |
| **55-59y** | 1.0000 | 1.0147 | 1.0599 | 1.1235 |
| **60-64y** | 1.0000 | 1.0147 | 1.0599 | 1.1235 |
| **65-69y** | 1.0000 | 1.0147 | 1.0599 | 1.1235 |
| **70-74y** | 1.0000 | 1.0147 | 1.0599 | 1.1235 |
| **75-79y** | 1.0000 | 1.0147 | 1.0599 | 1.1235 |
| **>=80y** | 1.0000 | 1.0147 | 1.0599 | 1.1235 |
|  |  |  |  |  |
| **Women** |  |  |  |  |
| **30-34y** | 1.0000 | 1.0147 | 1.0599 | 1.1235 |
| **35-39y** | 1.0000 | 1.0147 | 1.0599 | 1.1235 |
| **40-44y** | 1.0000 | 1.0147 | 1.0599 | 1.1235 |
| **45-49y** | 1.0000 | 1.0147 | 1.0599 | 1.1235 |
| **50-54y** | 1.0000 | 1.0147 | 1.0599 | 1.1235 |
| **55-59y** | 1.0000 | 1.0147 | 1.0599 | 1.1235 |
| **60-64y** | 1.0000 | 1.0147 | 1.0599 | 1.1235 |
| **65-69y** | 1.0000 | 1.0147 | 1.0599 | 1.1235 |
| **70-74y** | 1.0000 | 1.0147 | 1.0599 | 1.1235 |
| **75-79y** | 1.0000 | 1.0147 | 1.0599 | 1.1235 |
| **>=80y** | 1.0000 | 1.0147 | 1.0599 | 1.1235 |

**Table 11. Final PARs by age group. sex and range of salt consumption for stroke- - Brazil. 2017**

|  | **<5g** | **5-8g** | **8-12g** | **>=12g** |
| --- | --- | --- | --- | --- |
| **M15-19** | 0.00000 | 0.01658 | 0.11866 | 0.06716 |
| **M20-24** | 0.00000 | 0.01658 | 0.11866 | 0.06716 |
| **M25-29** | 0.00000 | 0.01658 | 0.11866 | 0.06716 |
| **M30-34** | 0.00000 | 0.01484 | 0.11831 | 0.08110 |
| **M35-39** | 0.00000 | 0.01484 | 0.11831 | 0.08110 |
| **M40-44** | 0.00000 | 0.01484 | 0.11831 | 0.08110 |
| **M45-49** | 0.00000 | 0.01658 | 0.11725 | 0.06666 |
| **M50-54** | 0.00000 | 0.01658 | 0.11120 | 0.06269 |
| **M55-59** | 0.00000 | 0.01658 | 0.11120 | 0.06269 |
| **M60-64** | 0.00000 | 0.01954 | 0.08871 | 0.05175 |
| **M65-69** | 0.00000 | 0.01954 | 0.08871 | 0.05175 |
| **M70-74** | 0.00000 | 0.01954 | 0.07301 | 0.04165 |
| **M75-79** | 0.00000 | 0.01954 | 0.07301 | 0.04165 |
| **M>=80** | 0.00000 | 0.01954 | 0.04235 | 0.02314 |
|  |  |  |  |  |
| **F15-19** | 0.00000 | 0.01496 | 0.11201 | 0.04719 |
| **F20-24** | 0.00000 | 0.01496 | 0.11201 | 0.04719 |
| **F25-29** | 0.00000 | 0.01496 | 0.11201 | 0.04719 |
| **F30-34** | 0.00000 | 0.01339 | 0.11167 | 0.05724 |
| **F35-39** | 0.00000 | 0.01339 | 0.11167 | 0.05724 |
| **F40-44** | 0.00000 | 0.01339 | 0.11167 | 0.05724 |
| **F45-49** | 0.00000 | 0.01496 | 0.11067 | 0.04683 |
| **F50-54** | 0.00000 | 0.01416 | 0.10492 | 0.04399 |
| **F55-59** | 0.00000 | 0.01416 | 0.10492 | 0.04399 |
| **F60-64** | 0.00000 | 0.01456 | 0.08358 | 0.03618 |
| **F65-69** | 0.00000 | 0.01456 | 0.08358 | 0.03618 |
| **F70-74** | 0.00000 | 0.01194 | 0.06872 | 0.02903 |
| **F75-79** | 0.00000 | 0.01194 | 0.06872 | 0.02903 |
| **F>=80** | 0.00000 | 0.00689 | 0.03978 | 0.01603 |

**Table 12. Final PARs by age group. sex and range of salt consumption for hypertensive disease- - Brazil. 2017**

|  | **<5g** | **5-8g** | **8-12g** | **>=12g** |
| --- | --- | --- | --- | --- |
| **M15-19** | 0.00000 | 0.00773 | 0.17331 | 0.10576 |
| **M20-24** | 0.00000 | 0.00773 | 0.17331 | 0.10576 |
| **M25-29** | 0.00000 | 0.00773 | 0.17331 | 0.10576 |
| **M30-34** | 0.00000 | 0.00691 | 0.17283 | 0.12661 |
| **M35-39** | 0.00000 | 0.00691 | 0.17283 | 0.12661 |
| **M40-44** | 0.00000 | 0.00691 | 0.17283 | 0.12661 |
| **M45-49** | 0.00000 | 0.00773 | 0.17139 | 0.10499 |
| **M50-54** | 0.00000 | 0.00751 | 0.17139 | 0.10499 |
| **M55-59** | 0.00000 | 0.00751 | 0.17139 | 0.10499 |
| **M60-64** | 0.00000 | 0.00787 | 0.15739 | 0.10116 |
| **M65-69** | 0.00000 | 0.00787 | 0.15739 | 0.10116 |
| **M70-74** | 0.00000 | 0.00652 | 0.15739 | 0.10116 |
| **M75-79** | 0.00000 | 0.00652 | 0.15739 | 0.10116 |
| **M>=80** | 0.00000 | 0.00510 | 0.15739 | 0.10116 |
|  |  |  |  |  |
| **F15-19** | 0.00000 | 0.01041 | 0.16417 | 0.07523 |
| **F20-24** | 0.00000 | 0.01041 | 0.16417 | 0.07523 |
| **F25-29** | 0.00000 | 0.01041 | 0.16417 | 0.07523 |
| **F30-34** | 0.00000 | 0.00932 | 0.16371 | 0.09068 |
| **F35-39** | 0.00000 | 0.00932 | 0.16371 | 0.09068 |
| **F40-44** | 0.00000 | 0.00932 | 0.16371 | 0.09068 |
| **F45-49** | 0.00000 | 0.01041 | 0.16232 | 0.07467 |
| **F50-54** | 0.00000 | 0.01012 | 0.16232 | 0.07467 |
| **F55-59** | 0.00000 | 0.01012 | 0.16232 | 0.07467 |
| **F60-64** | 0.00000 | 0.01061 | 0.14893 | 0.07185 |
| **F65-69** | 0.00000 | 0.01061 | 0.14893 | 0.07185 |
| **F70-74** | 0.00000 | 0.00879 | 0.14893 | 0.07185 |
| **F75-79** | 0.00000 | 0.00879 | 0.14893 | 0.07185 |
| **F>=80** | 0.00000 | 0.00689 | 0.14893 | 0.07185 |

**Table 13. Final PARs by age group. sex and range of salt consumption for heart failure- - Brazil. 2017**

|  | **<5g** | **5-8g** | **8-12g** | **>=12g** |
| --- | --- | --- | --- | --- |
| **M15-19** | 0.00000 | 0.00687 | 0.07451 | 0.03971 |
| **M20-24** | 0.00000 | 0.00687 | 0.07451 | 0.03971 |
| **M25-29** | 0.00000 | 0.00687 | 0.07451 | 0.03971 |
| **M30-34** | 0.00000 | 0.00614 | 0.07428 | 0.04824 |
| **M35-39** | 0.00000 | 0.00614 | 0.07428 | 0.04824 |
| **M40-44** | 0.00000 | 0.00614 | 0.07428 | 0.04824 |
| **M45-49** | 0.00000 | 0.00687 | 0.07359 | 0.03940 |
| **M50-54** | 0.00000 | 0.00687 | 0.07359 | 0.03940 |
| **M55-59** | 0.00000 | 0.00687 | 0.07359 | 0.03940 |
| **M60-64** | 0.00000 | 0.00811 | 0.06693 | 0.03786 |
| **M65-69** | 0.00000 | 0.00811 | 0.06693 | 0.03786 |
| **M70-74** | 0.00000 | 0.00811 | 0.06693 | 0.03786 |
| **M75-79** | 0.00000 | 0.00811 | 0.06693 | 0.03786 |
| **M>=80** | 0.00000 | 0.00811 | 0.06693 | 0.03786 |
|  |  |  |  |  |
| **F15-19** | 0.00000 | 0.00926 | 0.07014 | 0.02766 |
| **F20-24** | 0.00000 | 0.00926 | 0.07014 | 0.02766 |
| **F25-29** | 0.00000 | 0.00926 | 0.07014 | 0.02766 |
| **F30-34** | 0.00000 | 0.00828 | 0.06992 | 0.03369 |
| **F35-39** | 0.00000 | 0.00828 | 0.06992 | 0.03369 |
| **F40-44** | 0.00000 | 0.00828 | 0.06992 | 0.03369 |
| **F45-49** | 0.00000 | 0.00926 | 0.06926 | 0.02744 |
| **F50-54** | 0.00000 | 0.00926 | 0.06926 | 0.02744 |
| **F55-59** | 0.00000 | 0.00926 | 0.06926 | 0.02744 |
| **F60-64** | 0.00000 | 0.01093 | 0.06297 | 0.02636 |
| **F65-69** | 0.00000 | 0.01093 | 0.06297 | 0.02636 |
| **F70-74** | 0.00000 | 0.01093 | 0.06297 | 0.02636 |
| **F75-79** | 0.00000 | 0.01093 | 0.06297 | 0.02636 |
| **F>=80** | 0.00000 | 0.01093 | 0.06297 | 0.02636 |

**Table 14. Final PARs by age group. sex and range of salt consumption for pulmonary embolism- - Brazil. 2017**

|  | **<5g** | **5-8g** | **8-12g** | **>=12g** |
| --- | --- | --- | --- | --- |
| **M15-19** | 0.00000 | 0.00354 | 0.03885 | 0.01973 |
| **M20-24** | 0.00000 | 0.00354 | 0.03885 | 0.01973 |
| **M25-29** | 0.00000 | 0.00354 | 0.03885 | 0.01973 |
| **M30-34** | 0.00000 | 0.00316 | 0.03873 | 0.02408 |
| **M35-39** | 0.00000 | 0.00316 | 0.03873 | 0.02408 |
| **M40-44** | 0.00000 | 0.00316 | 0.03873 | 0.02408 |
| **M45-49** | 0.00000 | 0.00354 | 0.03835 | 0.01957 |
| **M50-54** | 0.00000 | 0.00354 | 0.03835 | 0.01957 |
| **M55-59** | 0.00000 | 0.00354 | 0.03835 | 0.01957 |
| **M60-64** | 0.00000 | 0.00418 | 0.03476 | 0.01879 |
| **M65-69** | 0.00000 | 0.00418 | 0.03476 | 0.01879 |
| **M70-74** | 0.00000 | 0.00418 | 0.03476 | 0.01879 |
| **M75-79** | 0.00000 | 0.00418 | 0.03476 | 0.01879 |
| **M>=80** | 0.00000 | 0.00418 | 0.03476 | 0.01879 |
|  |  |  |  |  |
| **F15-19** | 0.00000 | 0.00478 | 0.03649 | 0.01366 |
| **F20-24** | 0.00000 | 0.00478 | 0.03649 | 0.01366 |
| **F25-29** | 0.00000 | 0.00478 | 0.03649 | 0.01366 |
| **F30-34** | 0.00000 | 0.00427 | 0.03637 | 0.01669 |
| **F35-39** | 0.00000 | 0.00427 | 0.03637 | 0.01669 |
| **F40-44** | 0.00000 | 0.00427 | 0.03637 | 0.01669 |
| **F45-49** | 0.00000 | 0.00478 | 0.03602 | 0.01355 |
| **F50-54** | 0.00000 | 0.00478 | 0.03602 | 0.01355 |
| **F55-59** | 0.00000 | 0.00478 | 0.03602 | 0.01355 |
| **F60-64** | 0.00000 | 0.00565 | 0.03264 | 0.01300 |
| **F65-69** | 0.00000 | 0.00565 | 0.03264 | 0.01300 |
| **F70-74** | 0.00000 | 0.00565 | 0.03264 | 0.01300 |
| **F75-79** | 0.00000 | 0.00565 | 0.03264 | 0.01300 |
| **F>=80** | 0.00000 | 0.00565 | 0.03264 | 0.01300 |

**Table 15. Final PARs by age group. sex and range of salt consumption for Aortic aneurysm- - Brazil. 2017**

|  | **<5g** | **5-8g** | **8-12g** | **>=12g** |
| --- | --- | --- | --- | --- |
| **M15-19** | 0.00000 | 0.00773 | 0.07023 | 0.03721 |
| **M20-24** | 0.00000 | 0.00773 | 0.07023 | 0.03721 |
| **M25-29** | 0.00000 | 0.00773 | 0.07023 | 0.03721 |
| **M30-34** | 0.00000 | 0.00691 | 0.07001 | 0.04523 |
| **M35-39** | 0.00000 | 0.00691 | 0.07001 | 0.04523 |
| **M40-44** | 0.00000 | 0.00691 | 0.07001 | 0.04523 |
| **M45-49** | 0.00000 | 0.00773 | 0.06935 | 0.03692 |
| **M50-54** | 0.00000 | 0.00751 | 0.06935 | 0.03692 |
| **M55-59** | 0.00000 | 0.00751 | 0.06935 | 0.03692 |
| **M60-64** | 0.00000 | 0.00787 | 0.06305 | 0.03547 |
| **M65-69** | 0.00000 | 0.00787 | 0.06305 | 0.03547 |
| **M70-74** | 0.00000 | 0.00652 | 0.06305 | 0.03547 |
| **M75-79** | 0.00000 | 0.00652 | 0.06305 | 0.03547 |
| **M>=80** | 0.00000 | 0.00510 | 0.06305 | 0.03547 |
|  |  |  |  |  |
| **F15-19** | 0.00000 | 0.01041 | 0.06609 | 0.02590 |
| **F20-24** | 0.00000 | 0.01041 | 0.06609 | 0.02590 |
| **F25-29** | 0.00000 | 0.01041 | 0.06609 | 0.02590 |
| **F30-34** | 0.00000 | 0.00932 | 0.06588 | 0.03156 |
| **F35-39** | 0.00000 | 0.00932 | 0.06588 | 0.03156 |
| **F40-44** | 0.00000 | 0.00932 | 0.06588 | 0.03156 |
| **F45-49** | 0.00000 | 0.01041 | 0.06526 | 0.02569 |
| **F50-54** | 0.00000 | 0.01012 | 0.06526 | 0.02569 |
| **F55-59** | 0.00000 | 0.01012 | 0.06526 | 0.02569 |
| **F60-64** | 0.00000 | 0.01061 | 0.05931 | 0.02467 |
| **F65-69** | 0.00000 | 0.01061 | 0.05931 | 0.02467 |
| **F70-74** | 0.00000 | 0.00879 | 0.05931 | 0.02467 |
| **F75-79** | 0.00000 | 0.00879 | 0.05931 | 0.02467 |
| **F>=80** | 0.00000 | 0.00689 | 0.05931 | 0.02467 |

**Table 16. Final PARs by age group. sex and range of salt consumption for rheumatic heart disease- - Brazil. 2017**

|  | **<5g** | **5-8g** | **8-12g** | **>=12g** |
| --- | --- | --- | --- | --- |
| **M15-19** | 0.00000 | 0.00324 | 0.03564 | 0.01802 |
| **M20-24** | 0.00000 | 0.00324 | 0.03564 | 0.01802 |
| **M25-29** | 0.00000 | 0.00324 | 0.03564 | 0.01802 |
| **M30-34** | 0.00000 | 0.00290 | 0.03552 | 0.02200 |
| **M35-39** | 0.00000 | 0.00290 | 0.03552 | 0.02200 |
| **M40-44** | 0.00000 | 0.00290 | 0.03552 | 0.02200 |
| **M45-49** | 0.00000 | 0.00324 | 0.03517 | 0.01788 |
| **M50-54** | 0.00000 | 0.00324 | 0.03517 | 0.01788 |
| **M55-59** | 0.00000 | 0.00324 | 0.03517 | 0.01788 |
| **M60-64** | 0.00000 | 0.00383 | 0.03187 | 0.01716 |
| **M65-69** | 0.00000 | 0.00383 | 0.03187 | 0.01716 |
| **M70-74** | 0.00000 | 0.00383 | 0.03187 | 0.01716 |
| **M75-79** | 0.00000 | 0.00383 | 0.03187 | 0.01716 |
| **M>=80** | 0.00000 | 0.00383 | 0.03187 | 0.01716 |
|  |  |  |  |  |
| **F15-19** | 0.00000 | 0.00438 | 0.03346 | 0.01247 |
| **F20-24** | 0.00000 | 0.00438 | 0.03346 | 0.01247 |
| **F25-29** | 0.00000 | 0.00438 | 0.03346 | 0.01247 |
| **F30-34** | 0.00000 | 0.00391 | 0.03335 | 0.01524 |
| **F35-39** | 0.00000 | 0.00391 | 0.03335 | 0.01524 |
| **F40-44** | 0.00000 | 0.00391 | 0.03335 | 0.01524 |
| **F45-49** | 0.00000 | 0.00438 | 0.03303 | 0.01237 |
| **F50-54** | 0.00000 | 0.00438 | 0.03303 | 0.01237 |
| **F55-59** | 0.00000 | 0.00438 | 0.03303 | 0.01237 |
| **F60-64** | 0.00000 | 0.00517 | 0.02992 | 0.01187 |
| **F65-69** | 0.00000 | 0.00517 | 0.02992 | 0.01187 |
| **F70-74** | 0.00000 | 0.00517 | 0.02992 | 0.01187 |
| **F75-79** | 0.00000 | 0.00517 | 0.02992 | 0.01187 |
| **F>=80** | 0.00000 | 0.00517 | 0.02992 | 0.01187 |

**Table 17. Attributable hospitalization costs (US$) by age group and sex for coronary heart disease (CHD), stroke, hypertension disease (HD), heat failure (HF), aortic aneurysm (AA), pulmonary embolism (PE) and rheumatic heart disease (RHD)- - Brazil. 2017**

|  | CHD | Stroke | HD | HF | AA | PE | RHD | TOTAL |
| --- | --- | --- | --- | --- | --- | --- | --- | --- |
| Men |  |  |  |  |  |  |  |  |
| M15-19 | 11,761.67 | 120,935.45 | 17,738.02 | 48,833.26 | 1,888.56 | 30,629.04 | 12,655.16 | 244,441.15 |
| M20-24 | 24,809.96 | 155,264.42 | 29,144.94 | 43,842.25 | 3,792.93 | 39,436.53 | 16,070.00 | 312,361.02 |
| M25-29 | 53,595.09 | 177,346.55 | 25,367.20 | 58,928.55 | 2,861.97 | 47,352.27 | 21,305.44 | 386,757.08 |
| M30-34 | 161,407.97 | 261,755.85 | 42,859.13 | 96,820.80 | 8,004.08 | 52,319.43 | 32,122.34 | 655,289.59 |
| M35-39 | 400,151.59 | 400,756.75 | 87,539.67 | 139,555.77 | 6,167.51 | 77,755.49 | 44,687.33 | 1,156,614.11 |
| M40-44 | 940,593.21 | 578,988.80 | 113,570.71 | 247,612.94 | 8,545.63 | 109,502.67 | 45,109.74 | 2,043,923.70 |
| M45-49 | 1,914,781.35 | 809,487.60 | 128,787.32 | 326,603.68 | 4,783.48 | 145,723.60 | 53,166.17 | 3,383,333.21 |
| M50-54 | 3,467,709.15 | 1,162,256.45 | 141,573.56 | 513,233.42 | 7,514.78 | 223,927.47 | 59,889.90 | 5,576,104.72 |
| M55-59 | 4,777,804.47 | 1,406,567.86 | 160,182.84 | 748,602.23 | 12,235.34 | 333,176.17 | 74,979.36 | 7,513,548.28 |
| M60-64 | 4,651,718.40 | 1,421,748.05 | 163,527.89 | 847,248.21 | 13,271.22 | 486,832.28 | 77,425.31 | 7,661,771.36 |
| M65-69 | 4,193,968.28 | 1,434,779.94 | 143,020.49 | 905,835.59 | 9,681.07 | 590,925.43 | 67,762.02 | 7,345,972.81 |
| M70-74 | 2,600,778.29 | 1,084,372.82 | 112,711.44 | 807,577.18 | 7,547.16 | 483,395.52 | 44,872.03 | 5,141,254.43 |
| M75-79 | 1,651,617.93 | 877,766.04 | 99,464.53 | 703,247.98 | 8,773.04 | 390,460.77 | 32,501.11 | 3,763,831.40 |
| M80+ | 855,052.76 | 645,112.54 | 118,447.51 | 894,750.71 | 8,609.25 | 263,664.48 | 9,002.57 | 2,794,639.83 |
| **M Total** | **25,705,750.09** | **10,537,139.10** | **1,383,935.25** | **6,382,692.57** | **103,676.01** | **3,275,101.15** | **591,548.49** | **47,979,842.67** |
|  |  |  |  |  |  |  |  |  |
| Women |  |  |  |  |  |  |  |  |
| F15-19 | 7,770.64 | 53,173.37 | 20,833.53 | 25,390.64 | 2,456.31 | 9,223.93 | 9,273.38 | 128,121.79 |
| F20-24 | 14,172.82 | 89,198.61 | 20,872.93 | 29,658.65 | 4,893.38 | 10,772.55 | 17,807.17 | 187,376.11 |
| F25-29 | 24,221.35 | 149,686.19 | 30,621.26 | 39,619.50 | 6,422.50 | 17,382.62 | 28,657.62 | 296,611.04 |
| F30-34 | 59,102.23 | 234,422.65 | 53,112.82 | 62,814.65 | 7,278.35 | 25,194.16 | 42,845.94 | 484,770.79 |
| F35-39 | 172,486.78 | 416,407.94 | 61,934.36 | 107,126.36 | 10,242.89 | 42,140.60 | 62,336.17 | 872,675.12 |
| F40-44 | 391,177.46 | 652,056.79 | 99,327.80 | 135,505.50 | 9,838.93 | 53,119.92 | 74,097.26 | 1,415,123.65 |
| F45-49 | 811,456.25 | 927,905.12 | 90,003.54 | 222,217.58 | 12,417.09 | 65,648.35 | 77,511.08 | 2,207,159.01 |
| F50-54 | 1,307,793.53 | 1,140,701.64 | 115,415.52 | 349,861.43 | 10,219.47 | 97,918.79 | 93,662.58 | 3,115,572.98 |
| F55-59 | 2,032,060.69 | 1,214,735.17 | 117,362.03 | 477,348.43 | 11,445.50 | 147,872.12 | 86,954.42 | 4,087,778.37 |
| F60-64 | 2,101,361.89 | 1,036,103.77 | 130,998.46 | 548,983.94 | 13,803.29 | 167,581.73 | 71,462.48 | 4,070,295.56 |
| F65-69 | 2,092,544.50 | 1,086,778.44 | 145,690.06 | 623,060.60 | 12,061.67 | 205,940.01 | 57,123.61 | 4,223,198.89 |
| F70-74 | 1,472,924.82 | 796,600.17 | 129,065.49 | 644,997.12 | 13,143.22 | 214,210.28 | 46,813.71 | 3,317,754.80 |
| F75-79 | 1,049,651.40 | 765,640.32 | 101,789.42 | 621,279.84 | 13,261.73 | 153,764.30 | 22,062.60 | 2,727,449.60 |
| F80+ | 668,529.57 | 612,990.37 | 168,653.45 | 1,076,078.25 | 20,633.76 | 107,766.28 | 8,295.22 | 2,662,946.89 |
| **F Total** | **12,205,253.92** | **9,176,400.56** | **1,285,680.66** | **4,963,942.50** | **148,118.09** | **1,318,535.64** | **698,903.23** | **29,796,834.60** |

**Table 18. Attributable outpatient costs (US$) by age group and sex for coronary heart disease (CHD), stroke, hypertension disease (HD), heat failure (HF), aortic aneurysm (AA), pulmonary embolism (PE) and rheumatic heart disease (RHD)- - Brazil. 2017**

|  | **CHD** | **Stroke** | **HD** | **HF** | **AA** | **PE** | **RHD** | **TOTAL** |
| --- | --- | --- | --- | --- | --- | --- | --- | --- |
| Men |  |  |  |  |  |  |  |  |
| M15-19 | 3319 | 13242 | 5386 | 1328 | 42 | 29 | 75 | 23422 |
| M20-24 | 4790 | 14806 | 4634 | 2226 | 119 | 77 | 150 | 26802 |
| M25-29 | 6403 | 14796 | 5146 | 2818 | 234 | 95 | 165 | 29656 |
| M30-34 | 15934 | 20545 | 8618 | 2936 | 284 | 95 | 155 | 48567 |
| M35-39 | 37143 | 28475 | 13633 | 4389 | 283 | 375 | 335 | 84633 |
| M40-44 | 83159 | 38793 | 19126 | 4775 | 304 | 332 | 607 | 147097 |
| M45-49 | 148403 | 62121 | 25836 | 6872 | 265 | 510 | 1097 | 245103 |
| M50-54 | 252209 | 84764 | 39818 | 9858 | 315 | 660 | 1365 | 388988 |
| M55-59 | 362746 | 113646 | 53569 | 13870 | 377 | 1171 | 1778 | 547157 |
| M60-64 | 359742 | 112105 | 59867 | 14541 | 227 | 2252 | 1804 | 550539 |
| M65-69 | 324402 | 116330 | 58118 | 14836 | 284 | 1962 | 1501 | 517433 |
| M70-74 | 197977 | 76621 | 45731 | 13280 | 256 | 1598 | 1296 | 336758 |
| M75-79 | 121788 | 62366 | 30310 | 8620 | 247 | 1695 | 828 | 225854 |
| M80+ | 57885 | 37782 | 25443 | 10230 | 251 | 1234 | 434 | 133258 |
| M Total | 1975900 | 796390 | 395235 | 110578 | 3487 | 12086 | 11590 | 3305267 |
|  |  |  |  |  |  |  |  |  |
| Women |  |  |  |  |  |  |  |  |
| F15-19 | 2051 | 7078 | 5237 | 1782 | 27 | 13 | 139 | 16.327 |
| F20-24 | 2675 | 9621 | 5395 | 1137 | 51 | 123 | 61 | 19.062 |
| F25-29 | 5375 | 12244 | 8005 | 1015 | 39 | 105 | 248 | 27.030 |
| F30-34 | 11207 | 20408 | 12337 | 2007 | 69 | 81 | 365 | 46.475 |
| F35-39 | 28616 | 32943 | 19400 | 3426 | 80 | 190 | 508 | 85.163 |
| F40-44 | 70129 | 41313 | 27808 | 4330 | 194 | 508 | 875 | 145.157 |
| F45-49 | 135280 | 56248 | 39830 | 5826 | 140 | 945 | 1.166 | 239.434 |
| F50-54 | 219286 | 74035 | 56682 | 8538 | 170 | 1.061 | 1.518 | 361.290 |
| F55-59 | 293793 | 86368 | 68327 | 13226 | 352 | 1.432 | 1.840 | 465.338 |
| F60-64 | 287991 | 71617 | 71335 | 14273 | 175 | 2.471 | 1.908 | 449.771 |
| F65-69 | 268648 | 75496 | 68157 | 13455 | 207 | 3.205 | 1.401 | 430.569 |
| F70-74 | 168878 | 51082 | 56635 | 10571 | 120 | 3.063 | 1.042 | 291.391 |
| F75-79 | 105591 | 43170 | 38028 | 8066 | 98 | 2.181 | 619 | 197.754 |
| F80+ | 52703 | 33656 | 33955 | 6556 | 117 | 1.860 | 410 | 129.258 |
| F Total | 1/652223 | 615277 | 511131 | 94209 | 1.839 | 17.238 | 12.100 | 2.904.018 |

**Table 19. Attributable Years of Life Lost by age group and sex for coronary heart disease (CHD), stroke, hypertension disease (HD), heat failure (HF), aortic aneurysm (AA), pulmonary embolism (PE) and rheumatic heart disease (RHD)- - Brazil. 2017**

|  | Standard LE | CHD | Stroke | HD | HF | AA | PE | RHD | CVD |
| --- | --- | --- | --- | --- | --- | --- | --- | --- | --- |
| Men |  |  |  |  |  |  |  |  |  |
| 15-19y | 57 | 545 | 320 | 60 | 87 | 32 | 10 | 34 | 1,089 |
| 20-24y | 53 | 754 | 815 | 475 | 140 | 69 | 34 | 40 | 2,326 |
| 25-29y | 48 | 950 | 1,116 | 505 | 159 | 93 | 55 | 61 | 2,940 |
| 30-34y | 44 | 1,670 | 2,049 | 1,018 | 225 | 142 | 77 | 56 | 5,238 |
| 35-39y | 39 | 2,818 | 3,587 | 1,885 | 427 | 261 | 122 | 64 | 9,163 |
| 40-44y | 35 | 4,861 | 6,342 | 2,608 | 645 | 339 | 157 | 62 | 15,014 |
| 45-49y | 31 | 6,427 | 9,091 | 4,651 | 815 | 491 | 152 | 64 | 21,690 |
| 50-54y | 27 | 8,658 | 12,968 | 7,192 | 1,156 | 627 | 159 | 95 | 30,856 |
| 55-59y | 23 | 10,815 | 15,801 | 8,676 | 1,688 | 762 | 193 | 88 | 38,022 |
| 60-64y | 19 | 11,283 | 14,695 | 9,872 | 2,074 | 906 | 179 | 79 | 39,086 |
| 65-69y | 16 | 12,104 | 13,566 | 10,061 | 2,196 | 1,046 | 171 | 82 | 39,226 |
| 70-74y | 13 | 9,772 | 9,489 | 9,258 | 2,137 | 817 | 151 | 57 | 31,681 |
| 75-79y | 10 | 8,742 | 7,167 | 7,943 | 2,095 | 677 | 121 | 45 | 26,790 |
| 80+ | 9 | 10,613 | 8,607 | 19,587 | 4,942 | 821 | 267 | 66 | 44,903 |
| **Total** |  | **90,011** | **105,613** | **83,791** | **18,786** | **7,082** | **1,849** | **890** | **308,022** |
|  |  |  |  |  |  |  |  |  |  |
| Women |  | CHD | Stroke | HD | HF | AA | PE | RHD | CVD |
| 15-19y | 64 | 342 | 78 | 96 | 17 | 10 | 23 | 22 | 588 |
| 20-24y | 59 | 542 | 168 | 178 | 81 | 25 | 41 | 34 | 1,070 |
| 25-29y | 54 | 836 | 321 | 285 | 108 | 34 | 58 | 58 | 1,700 |
| 30-34y | 49 | 1,524 | 650 | 610 | 122 | 28 | 104 | 88 | 3,126 |
| 35-39y | 45 | 2,766 | 1,379 | 1,251 | 211 | 127 | 126 | 112 | 5,973 |
| 40-44y | 40 | 4,329 | 2,580 | 2,341 | 355 | 171 | 159 | 102 | 10,037 |
| 45-49y | 35 | 6,017 | 4,015 | 3,473 | 563 | 208 | 188 | 142 | 14,606 |
| 50-54y | 31 | 7,017 | 5,350 | 4,716 | 795 | 301 | 164 | 175 | 18,518 |
| 55-59y | 27 | 7,845 | 6,891 | 6,222 | 1,079 | 363 | 170 | 177 | 22,746 |
| 60-64y | 23 | 7,948 | 7,598 | 7,551 | 1,493 | 513 | 180 | 147 | 25,430 |
| 65-69y | 19 | 8,942 | 8,075 | 8,196 | 1,695 | 589 | 201 | 135 | 27,833 |
| 70-74y | 15 | 7,902 | 6,658 | 8,726 | 2,004 | 580 | 215 | 101 | 26,187 |
| 75-79y | 12 | 8,514 | 6,116 | 9,307 | 2,208 | 525 | 193 | 76 | 26,940 |
| 80+ | 10 | 14,995 | 34,622 | 33,129 | 7,798 | 853 | 583 | 128 | 92,110 |
| **Total** |  | **79517** | **84,503** | **86,083** | **18,529** | **4,328** | **2,404** | **1,497** | **276,861** |

**Table 20. Attributable Years of Productive Life Lost and productivity losses (US$) by age group and sex - - Brazil. 2017**

|  | **Mean age** | **Life expectancy at age** | **Month wage US$** | **Workplace participation** | **Pension age** | **YPLL** | **Economic loss (US$)** |
| --- | --- | --- | --- | --- | --- | --- | --- |
| Men |  |  |  |  |  |  |  |
| 15-19y | 17.5 | 57.1 | 662.07 | 0.193 | 65 | 47.5 | 2,902,392.88 |
| 20-24y | 22.5 | 52.6 | 662.07 | 0.693 | 65 | 42.5 | 20,576,309.98 |
| 25-29y | 27.5 | 48.2 | 662.07 | 0.824 | 65 | 37.5 | 28,317,374.84 |
| 30-34y | 32.5 | 43.8 | 662.07 | 0.824 | 65 | 32.5 | 45,729,544.39 |
| 35-39y | 37.5 | 39,3 | 662.07 | 0.824 | 65 | 27.5 | 71,431,102.02 |
| 40-44y | 42.5 | 35,0 | 662.07 | 0.731 | 65 | 22.5 | 90,600,779.36 |
| 45-49y | 47.5 | 30,7 | 662.07 | 0.731 | 65 | 17.5 | 109,911,376.64 |
| 50-54y | 52.5 | 26.6 | 662.07 | 0.731 | 65 | 12.5 | 122,265,163.79 |
| 55-59y | 57.5 | 22.7 | 662.07 | 0.731 | 65 | 7.5 | 100,585,429.59 |
| 60-64y | 62.5 | 19.0 | 662.07 | 0.077 | 65 | 2.5 | 4,120,975.43 |
| **Total** |  |  |  |  |  |  | **596,440,448.92** |
|  |  |  |  |  |  |  |  |
| Women |  |  |  |  |  |  |  |
| 15-19y | 17.5 | 63.9 | 662.07 | 0.193 | 60 | 42.5 | 1,347,224.01 |
| 20-24y | 22.5 | 59.1 | 662.07 | 0.693 | 60 | 37.5 | 7,981,464.85 |
| 25-29y | 27.5 | 54.2 | 662.07 | 0.824 | 60 | 32.5 | 13,502,256.89 |
| 30-34y | 32.5 | 49.4 | 662.07 | 0.824 | 60 | 27.5 | 21,840,778.98 |
| 35-39y | 37.5 | 44.7 | 662.07 | 0.824 | 60 | 22.5 | 35,758,757.78 |
| 40-44y | 42.5 | 40.0 | 662.07 | 0.731 | 60 | 17.5 | 43,815,698.33 |
| 45-49y | 47.5 | 35.4 | 662.07 | 0.731 | 60 | 12.5 | 48,641,052.19 |
| 50-54y | 52.5 | 30.9 | 662.07 | 0.731 | 60 | 7.5 | 40,023,877.76 |
| 55-59y | 57.5 | 26.6 | 662.07 | 0.731 | 60 | 2.5 | 18,007,607.49 |
| **Total** |  |  |  |  |  |  | **230,918,718.29** |
